# Prediction of colorectal cancer diagnosis based on circulating plasma proteins

**DOI:** 10.15252/emmm.201404873

**Published:** 2015-08-07

**Authors:** Silvia Surinova, Meena Choi, Sha Tao, Peter J Schüffler, Ching-Yun Chang, Timothy Clough, Kamil Vysloužil, Marta Khoylou, Josef Srovnal, Yansheng Liu, Mariette Matondo, Ruth Hüttenhain, Hendrik Weisser, Joachim M Buhmann, Marián Hajdúch, Hermann Brenner, Olga Vitek, Ruedi Aebersold

**Affiliations:** 1 Department of Biology, Institute of Molecular Systems Biology, ETH ZurichZurich, Switzerland; 2 Department of Statistics, Purdue UniversityWest Lafayette, IN, USA; 3 Division of Clinical Epidemiology and Aging Research, German Cancer Research Center (DKFZ)Heidelberg, Germany; 4 Department of Computer Science, Institute for Machine Learning, ETH ZurichZurich, Switzerland; 5 Department of Surgery, University Hospital in OlomoucOlomouc, Czech Republic; 6 Institute of Molecular and Translational Medicine, Faculty of Medicine and Dentistry, Palacký UniversityOlomouc, Czech Republic; 7 German Cancer Consortium (DKTK), German Cancer Research Center (DKFZ)Heidelberg, Germany; 8 Department of Computer Science, Purdue UniversityWest Lafayette, IN, USA; 9 College of Science and College of Computer and Information Science, Northeastern UniversityBoston, MA, USA; 10 Faculty of Science, University of ZurichZurich, Switzerland

**Keywords:** colorectal cancer, diagnostic protein biomarker, discovery-driven and targeted proteomics

## Abstract

Non-invasive detection of colorectal cancer with blood-based markers is a critical clinical need. Here we describe a phased mass spectrometry-based approach for the discovery, screening, and validation of circulating protein biomarkers with diagnostic value. Initially, we profiled human primary tumor tissue epithelia and characterized about 300 secreted and cell surface candidate glycoproteins. These candidates were then screened in patient systemic circulation to identify detectable candidates in blood plasma. An 88-plex targeting method was established to systematically monitor these proteins in two large and independent cohorts of plasma samples, which generated quantitative clinical datasets at an unprecedented scale. The data were deployed to develop and evaluate a five-protein biomarker signature for colorectal cancer detection.

See also: **S Surinova *et al*** (September 2015)

## Introduction

Sporadic colorectal cancer (CRC) can be effectively cured by surgical resection if detected at localized disease stages (Booth, 2007). The current CRC detection procedure typically employs the fecal occult blood test (FOBT) as a pre-selection test for further colonoscopic evaluation. FOBT, however, presents a limited accuracy for tumor detection because it is a nonspecific test for gastrointestinal bleeding. As a result, FOBT does not adequately detect subjects with CRC and suffers from a limited sensitivity (Bretthauer, 2011). It is desirable to develop novel diagnostic tests that can replace or complement FOBT and lead to more accurate disease detection rates.

Preferably, diagnostic tests should be non-invasive, measurable in commonly sampled clinical specimens such as blood plasma, and better separate true from false CRC instances than the FOBT. Blood plasma and the proteins it contains are an ideal source of biomarkers, since it represents the snapshot of a subject’s patho-physiological state at a given time (Anderson & Anderson, 2002). We hypothesize that the pathological processes of CRC lead to characteristic changes in the proteins released from the tumor into the bloodstream, representing a CRC-derived molecular signature in plasma (see also Surinova *et al*, 2015).

We have set off to characterize proteins associated with CRC that are detectable in patient’s systemic circulation and to develop a protein biomarker signature able to classify CRC and control cases at risk. We employed a phased mass spectrometry-based approach for the discovery, screening, and validation of circulating protein biomarkers with diagnostic value. Initially, we profiled human primary tumor tissues and characterized about 300 secreted and cell surface candidate glycoproteins. These candidates were then screened in patient’s systemic circulation to identify a refined set of candidates detectable in blood plasma. An 88-plex targeting method was established to systematically monitor these proteins in two large and independent cohorts of plasma samples, which generated quantitative clinical datasets at an unprecedented scale. The data were used to develop and evaluate a five-protein biomarker signature that predicted colorectal cancer with high accuracy.

## Results

### Phase 1: biomarker candidate discovery in tumor epithelia

**Figure 1:**
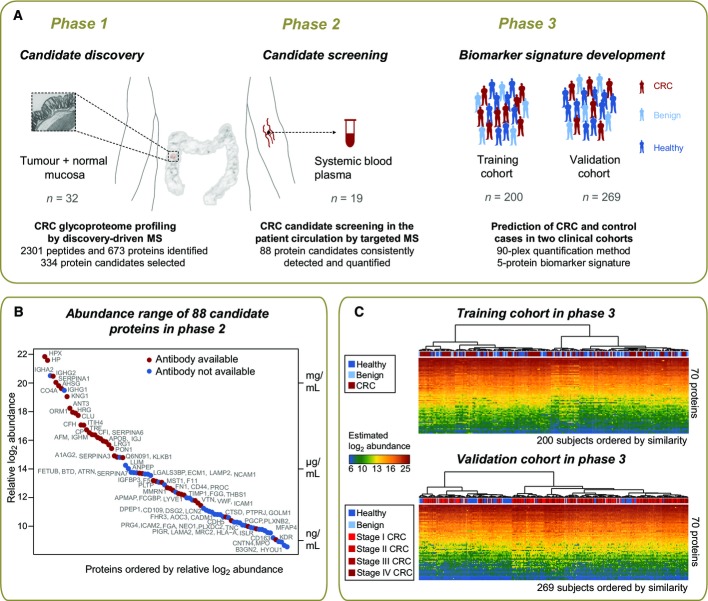
Phased proteomic workflow for the development of predictive biomarkers for colorectal cancer detection Biomarker candidates were first characterized in the tumor and normal tissue epithelia by discovery-driven mass spectrometric (MS) profiling of the glycoproteome and then screened in patient plasma by targeted MS via selected reaction monitoring (SRM). The detectable proteins in plasma were subjected to large-scale quantification across two clinical cohorts of samples comprised of subjects with CRC, subjects with benign conditions, and healthy subjects. The data were used to develop and validate a protein biomarker signature.

To maximize likelihood of identifying colorectal cancer (CRC) biomarkers in the circulation, a phased biomarker development pipeline was established (Fig1A). Human primary tumors together with adjacent normal mucosa were sampled from 16 subjects with CRC (Appendix Table S1) as the best suitable source of biomarkers. Tissue epithelia were manually dissected to enrich for cells of cancer origin and to obtain samples with maximally homogenous protein composition (Appendix Fig S1). To further enhance the capture of circulating proteins and to gain access to the lower abundant fraction of the plasma proteome, we selectively focused on glycoproteins which are typically cell surface and extracellular proteins prone to secretion or shedding and are representative of the vast majority of currently approved biomarkers (Zhang *et al*, 2007; Schiess *et al*, 2009).

The relative abundances of protein biomarker candidates detected and quantified in patient circulation were estimated on a relative scale using linear mixed modeling. The *y*-axis on the right side of the plot annotates the proteins with measured concentrations by immunoassays in plasma (Haab *et al*, 2005; Polanski & Anderson, 2007; Hortin *et al*, 2008). The proteins span a concentration range of 5–6 orders of magnitude. Red dots indicate proteins with available immunoassays in plasma, and blue dots indicate proteins without such immunoassays.

The generated quantification data are presented for the training and validation datasets, and the disease status of samples is labeled with red for CRC subjects, light blue for subjects with benign conditions, and dark blue for healthy subjects. Hierarchical clustering with Euclidian distance and Ward linkage was employed to cluster samples by similarity of protein abundance.

Epithelial lysates derived from 32 paired tumor and normal samples were therefore subjected to proteolysis, followed by solid-phase extraction of *N*-linked glycopeptides (Zhang *et al*, 2003). Purified *N*-glycosite peptides (de-glycosylated forms of peptides that are glycosylated in the native protein) were analyzed as duplicates or triplicates (as described in Materials and Methods) by high-resolution liquid chromatography tandem mass spectrometry (LC-MS/MS). In total, 74 LC-MS runs were acquired and led to the identification of 2,301 glycopeptides and 673 inferred glycoproteins (https://db.systemsbiology.net/sbeams/cgi/PeptideAtlas/buildDetails?atlas_build_id=374, or Table EV1). Prediction analysis of secondary protein structures annotated 73% of these proteins as prone to secretion, and 53% as containing at least one transmembrane domain. This is indicative of a strong enrichment for proteins of the circulatory system (Roth, 2002).

Peptide MS1-level features were quantified across all LC-MS runs to characterize proteins that were consistently changing in their abundance between tumor samples and their paired normal counterparts and to assess differential protein abundance across cancer progression. In total, 303 differentially abundant glycoproteins (adjusted *P* < 0.05, log_2_ FC cutoff ± 1.5) showed robust protein changes in CRC, irrespective of the individual clinical stages, distinct changes across disease progression, or specific differences between localized and metastatic CRC (Table EV2).

### Phase 2: screening of biomarker candidates in patient plasma

The hypothesis that secreted and cell surface glycoprotein candidates of CRC are destined to reach the circulation was tested in the screening phase (Fig1A) of the study. In this phase, the 303 glycoproteins identified as differentially abundant in CRC tissue were supplemented with 23 additional proteins identified in the tumor glycoproteome and being associated with cancer in the literature, as well as five biomarker candidates identified in other ongoing cancer biomarker studies (Table EV2) to test their detection in plasma.

Targeted mass spectrometry based on selected reaction monitoring (SRM) was employed to screen for tissue-derived candidates in *N*-glycosite samples enriched from plasma from 19 subjects with CRC. Subjects used in the screening phase partially overlapped with subjects employed in the discovery phase (Phase 1), as described in Appendix Table S1. Using the targeted approach, we detected 88 candidate proteins consistently in all plasma samples (Table EV3). While the dynamic range of the plasma proteome spreads over more than 10 orders of magnitude and poses major challenges to its comprehensive analysis (Anderson & Anderson, 2002), this study succeeded in detecting and quantifying candidate proteins covering 6 orders of magnitude in dynamic range. To our knowledge, this is currently the largest abundance range quantifiable in a single LC-MS analysis of plasma (Fig1B). When relating protein concentrations measured by immunoassays in plasma (Haab *et al*, 2005; Polanski & Anderson, 2007; Hortin *et al*, 2008) to plasma protein abundances determined in this study, it can be seen that the quantifications based on the two technologies are in a reasonable agreement. This is supported by the observation that the protein order sorted by immunoassay concentrations or mass spectrometry relative abundances was rather constant (Fig1B). Next, we examined the coverage of our biomarker candidates by immunoassays in plasma. From the literature, we know that immunoassays are only available for a very small portion of the proteome and this is also the case for the cell surface proteome (Bock *et al*, 2012). In our study, as many as half of the protein candidates were previously assayed by antibody-based technologies in plasma (red dots, Fig1B). Interestingly, it can be noted that, especially for the lower protein abundance range, immunoassays are nearly completely absent, and therefore, targeted mass spectrometry provides the means to quantify those candidates that could not be previously measured.

### Phase 3: development of a diagnostic biomarker signature

The biomarker candidates detected in plasma were then subjected to clinical evaluation in two independent cohorts of samples (*n* = 469) to ascertain biomarker candidates with diagnostic value (Table1). In both cohorts, the disease group was comprised of subjects with CRC and the control group included both subjects with benign lesions and healthy subjects. The first cohort—*the training cohort*—was designed to reflect an underlying population with CRC or at risk for CRC and was used for the discovery of a biomarker signature with diagnostic potential. It included healthy subjects (*n* = 66) and subjects with benign lesions (*n* = 34), and subjects with CRC (*n* = 100). The disease status of all subjects was confirmed by colonoscopy. The second cohort—*the validation cohort*—was comprised of healthy subjects (*n* = 50) and subjects with benign lesions (*n* = 17), and subjects with CRC (*n* = 202) at distinct clinical stages (stage I: *n* = 43, stage II: *n* = 58, stage III: *n* = 49, stage IV: *n* = 52). The validation cohort was conceived to test the discovered protein biomarker signature on independent samples and to evaluate the ability of the signature to classify the disease status with respect to clinical stage. Plasma samples were subjected to parallel *N*-glycoprotein extraction in a 96-well format, followed by targeted quantification of the candidate proteins by SRM. Candidate proteins, together with two protein standards, were combined into a 90-plex SRM method and used to profile the biomarker candidates over the plasma-enriched *N*-glycosite samples. The training and the validation cohorts were profiled separately and independently. Of the 88 biomarker candidates, 70 proteins were consistently quantified in both cohorts. This constitutes by far the largest clinical dataset measured by LC-MS to date (Fig1C).

**Table 1:**
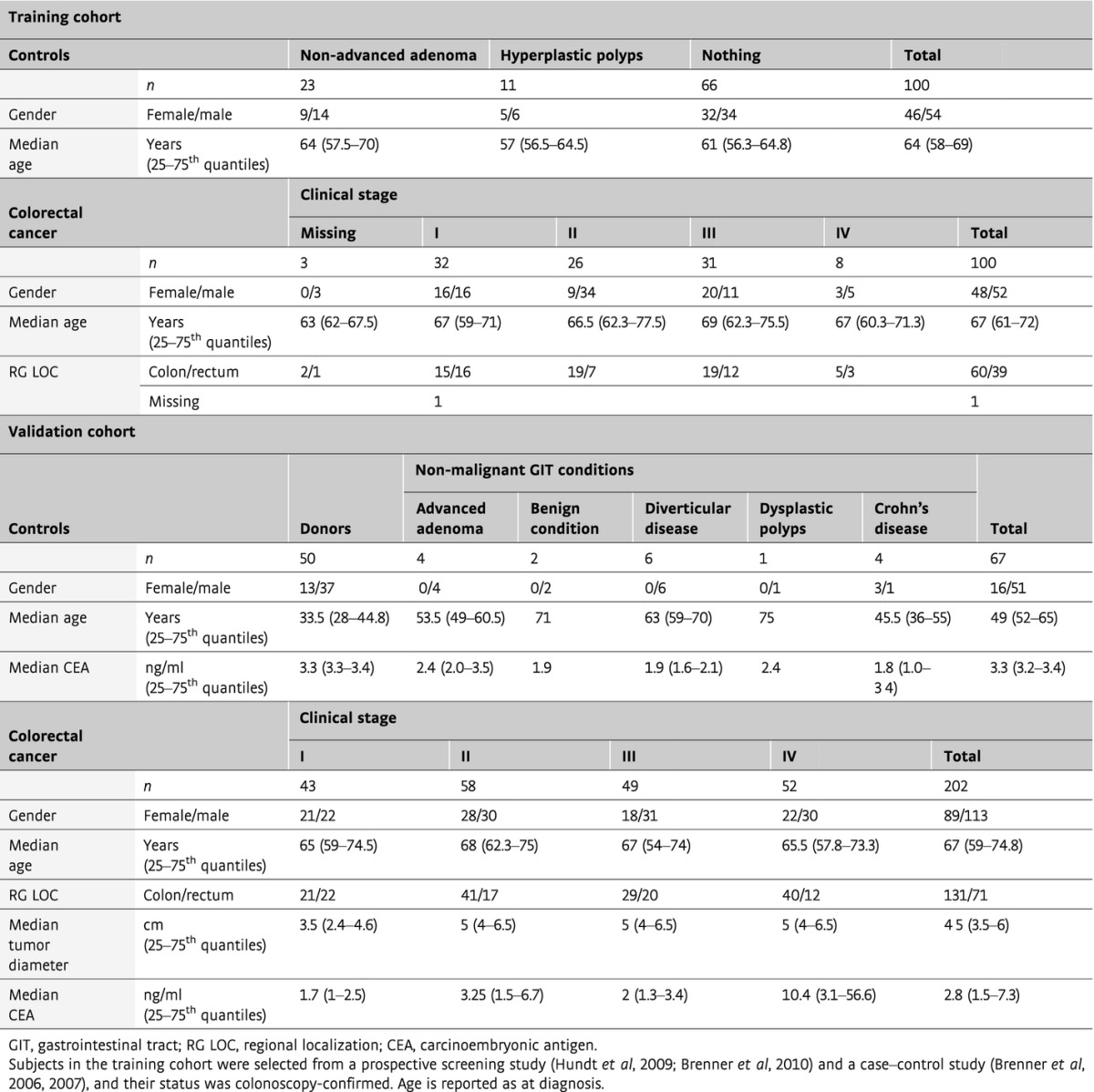
Characteristics of the study population according to diagnostic subgroup for the training and validation cohorts

**Figure 2:**
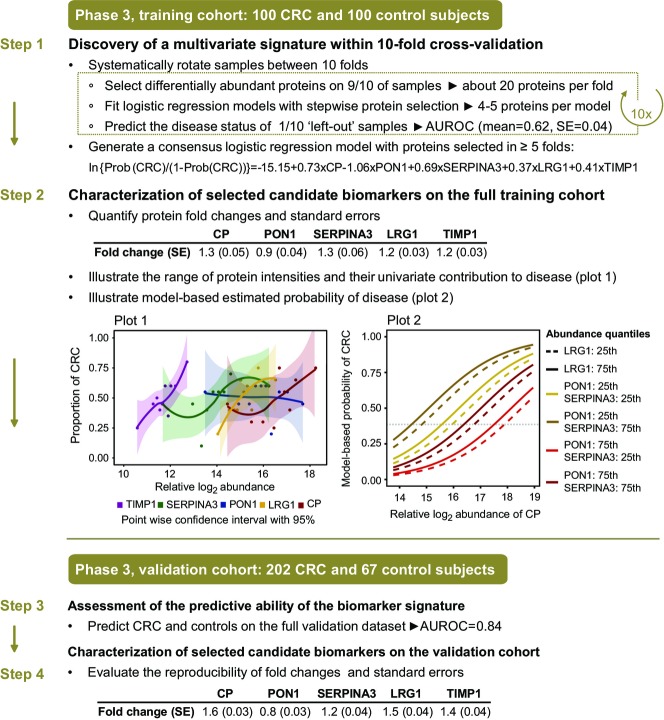
Protein biomarker signature development in phase 3 Step 1: A training cohort with 200 samples (100 CRC, 100 control subjects) was used to discover a predictive signature using multivariate logistic regression and 10-fold cross-validation. The parameters of the multivariate logistic regression used to evaluate the predictive ability of the signature are reported for the model. Step 2: The selected protein biomarkers were characterized on the full training cohort. Plot 1: For each protein in the signature, subjects were partitioned into 10 equal groups of relative protein abundance, and the proportion of subjects with CRC was calculated for each group and plotted as a function of relative protein abundance. Loess curves (Cleveland & Shyu, 1992) were fit to the points together with bands indicating 95% pointwise confidence intervals for the curves. Plot 2: model-based probabilities of CRC, estimated with the multivariate signature on the full training cohort. The probabilities were plotted as a function of relative abundance of CP. The colored lines correspond to probabilities at the 25^th^ or 75^th^ quantiles of relative abundance of LRG1, PON1, and SERPINA3. Dashed lines correspond to LRG1 fixed at the 25^th^ quantile, and solid lines indicate LRG1 fixed at the 75^th^ quantile of relative abundance. TIMP1 was fixed at the median value of relative abundance. Step 3: An independently acquired validation cohort was used to evaluate the accuracy of the protein biomarker signature. Step 4: Post-signature characterization of the selected biomarkers was also performed on the validation cohort in terms of fold change of differential abundance between the CRC and control groups, and the associated standard error. The fold changes and the standard errors reported in steps 2 and 4 were estimated using a linear mixed effect model (Choi *et al*, 2014) and transformed to the original scale using Delta method (Agresti, 2012) (FC_original_ = 2^log2^^FC^ and SE_original_ = SE log2 scale × *ln*(2)2^log2^^FC^).

To develop a diagnostic biomarker signature, we employed tenfold cross-validation on the training cohort (Fig2, Step 1). Within each fold, first a test of differential abundance between the CRC group and the controls was employed to narrow down the candidate list to a subset of significant proteins changing in abundance in CRC (*P*-value ≤ 0.05, FC cutoff ± 1.1) (Appendix Table S2A). Second, these proteins were used as input for logistic regression, and the most discriminative proteins were identified by stepwise selection. Third, their predictive accuracy was evaluated using the subjects left out in the cross-validation fold, and summarized with a ROC curve (Appendix Table S2B). The procedure was repeated 10 times by systematically leaving out different subjects. A consensus model was formed from proteins that were selected in at least 5 of the 10 repetitions. The consensus protein biomarker signature was comprised of ceruloplasmin (CP), serum paraoxonase/arylesterase 1 (PON1), serpin peptidase inhibitor, clade A (SERPINA3), leucine-rich alpha-2-glycoprotein (LRG1), and tissue inhibitor of metalloproteinases 1 (TIMP1). The parameters of the consensus model and the standard errors of these parameters based on the logistic regression model fit are reported in Appendix Fig S2.

To confirm the reproducibility of the protein biomarker signature, we repeated the procedure described above anew three times with different random partitions of the subjects into 10-folds and three more times with different random partitions of the subjects into eight-folds. In these repetitions, the proteins selected most frequently into the consensus models were highly overlapping with the proteins found above, and the prediction accuracy was also highly comparable (Appendix Table S3). Moreover, to compare the results with the optimum predictor on the training dataset, we enumerated all the protein combinations of up to five proteins in the training dataset by exhaustive search and evaluated the corresponding logistic regression models by their area under the ROC curve in 100-fold bootstrapped cross-validation (Efron & Tibshirani, 1993). The best models had a similar cross-validation performance. The proteins present were ranked by their frequency of occurrence among these models (Appendix Fig S3), and the top selections included the proteins in the protein biomarker signature above. Overall, these results confirm that the markers selected by the original procedure are robust to the specific choice of the parameters and of the folds.

Next, to fully take advantage of this large-scale dataset, we characterized the proteins in the selected biomarker signature above on the full training cohort. In the univariate analysis, an increase in protein abundance was associated with CRC for four out of the five proteins (Fig2, Step 2, Plot 1, for boxplots, see Appendix Fig S4A). PON1 showed the opposite trend. To illustrate the multivariate pattern of the protein biomarker signature, we plotted the predicted probability of CRC as a function of estimated log-abundance of CP, while fixing the estimated abundances of the other proteins to their quantiles (Fig2, Step 2, Plot 2). The highest probability of disease can be indeed achieved at the lower abundance quantile of PON1 and at the higher cutoffs of the other four proteins.

Given that the control group included healthy subjects with no lesions and subjects with benign lesions, the specificity of classification was evaluated separately for these two groups. In this analysis, the subjects were split into five-folds (instead of the original 10-folds) due to smaller-sized subgroups and the pseudomedian cross-validated performance of the respective subgroups was assessed. Sixty-two percent of subjects with no lesions and 57% of subjects with pre-lesions were accurately classified (Appendix Fig S5A). This shows that both groups were predicted with similar accuracy, which is in line with the 60% specificity of the complete control group. To investigate this important point of control group specificity further, a new set of advanced adenoma samples (*n* = 50) was included in the study at this stage. These samples were collected and measured as part of the training cohort, appropriately randomized and normalized to avoid bias. These new samples were not part of the protein biomarker signature development stage, as they represent intermediate lesions, and therefore can be viewed as a separate evaluation cohort. At this point, they were classified with the protein biomarker signature. Fifty-four percent of the subjects with advanced pre-lesions were correctly predicted (Appendix Fig S5B), which is similar but slightly inferior to the specificity seen for the complete control group. The observed drop in specificity highlights that these subjects represent an intermediate state of colorectal transformation.

**Figure 3:**
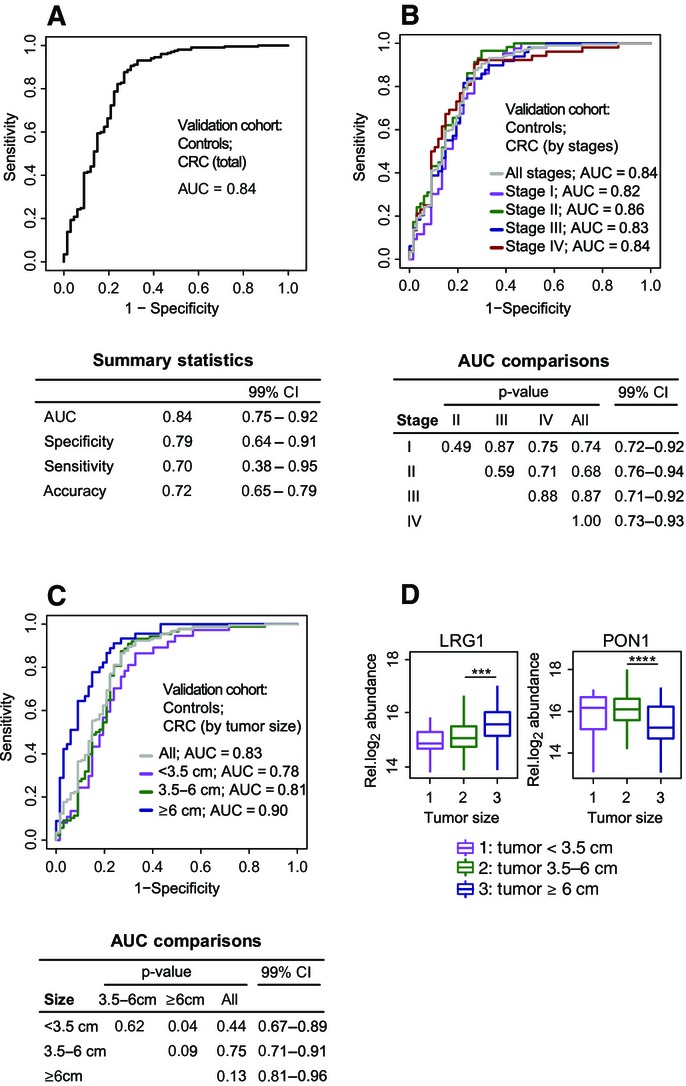
Evaluation of the protein biomarker signature on the independent validation cohort in phase 3 Detection of the disease status of subjects with CRC (*n* = 202) and controls (*n* = 67) was summarized in an ROC curve.

Finally, the protein biomarker signature was evaluated on the independent validation cohort. Each signature protein was first examined separately. For the five selected proteins, the fold changes of abundance between the CRC and the control groups, and the corresponding standard errors, were very well reproduced on the validation cohort (Fig2, Step 4, for boxplots, see Appendix Fig S4B). Of the five proteins, CP, TIMP1, and LRG1 demonstrated the highest areas under the ROC curves (Appendix Fig S6). The prediction of CRC and control subjects with the protein biomarker signature achieved an accuracy of 72% (Fig3A).

Stratified detection by clinical stage. The subjects with CRC were partitioned according to stage: CRC stage I (*n* = 43), CRC stage II (*n* = 58), CRC stage III (*n* = 48) and CRC stage IV (*n* = 52). Each group was discriminated separately against the controls (*n* = 67) using the same multivariate biomarker signature as in (A).

Stratified detection by tumor size. The subjects with CRC were partitioned according to tumor size: diameter < 3.5 cm (*n* = 37), diameter 3.4–6 cm (*n* = 88), and diameter ≥ 6 cm (*n* = 45). Each group was discriminated separately against the controls (*n* = 67) using the same multivariate biomarker signature as in (A).

Estimated relative protein abundance of the signature proteins in subjects with CRC, stratified by tumor size. The plotted relative log_2_ protein abundance represents a summarized value for each subject obtained from the linear model. Significant differences in abundance are labeled with asterisks (***P < 0.001, ****P < 0.0001). Data information: In all panels, a threshold of 0.386 was used to read specificity, sensitivity, and accuracy. Accuracy is defined as the proportion of true results in all the measurements [i.e. accuracy = (true positive + true negative)/(positive + negative)]. Pairwise tests of equality of means and of areas under the ROC curves were carried out with the *t*-test (in D) and using bootstrap cross-validation repeated 2,000 times to account for the non-independence of the curves in (B) and (C), respectively.

At this point, we examined whether age, a potential confounder, had any impact on the predictive ability of the protein biomarker signature. In the training cohort, the protein selection performed within 10-fold cross-validation with or without age adjustment of disease probability found that very similar proteins were selected in both cases. The difference between the respective AUCs was 2.9% (bootstrap-based *P*-value = 0.15). In the validation cohort, the difference between the model with or without age consideration was 5.2% (bootstrap-based *P*-value = 0.004). Although the difference in AUCs is statistically significant, in both cases similar AUC values were obtained (Appendix Fig S7).

In addition to the detection of CRC as one disease, we examined the ability of the protein biomarker signature to distinguish subjects with distinct stages of CRC from controls. The CRC subjects in the validation cohort were stratified according to the TNM system (Sobin, 2003), which represents the invasiveness in terms of tumor spread across the mucosal membrane. The results did not indicate a superior predictive ability of the protein biomarker signature for any particular disease stage (Fig3B). This showed that the protein biomarker signature can predict early and advanced CRC equally well.

Since the TNM-based staging of CRC does not account for the tumor extent, a smaller but more invasive tumor may be assigned to a more advanced stage than a larger but less invasive tumor. At the same time, larger tumors may secrete into circulation larger amounts of the protein biomarker and may therefore be easier to diagnose. To investigate the effect of the tumor size on the predictive ability of the protein biomarker signature, we assigned 170 CRC subjects in the validation cohort with recorded tumor size into three groups. Group 1 consisted of patients with tumor diameters smaller than < 3.5 cm (*n* = 37). Group 2 consisted of patients with tumors of a diameter between 3.5 and 6 cm (*n* = 88). Group 3 consisted of patients with large tumors of diameters equal to or larger than 6 cm (*n* = 45). As expected, the protein biomarker signature had a better predictive ability for subjects with large tumors than for subjects with smaller tumors (Fig3C). Univariate analysis showed higher levels of LRG1 in the circulation of subjects with larger tumors, pointing toward a specific protein secretion from the tumor (small versus medium tumors: *P* = 5.7e-2; medium versus large tumors: *P* = 7.7e-4, Fig3D). At the same time, and as expected from the differential abundance between disease and control groups (Fig2), smaller amounts of PON1 were detected in patients with larger tumors (small versus medium tumors: *P* = 1.6e-1; medium versus large tumors: *P* = 6.7e-5). In both cases, the trends were especially pronounced and highly significant when comparing tumors larger than 6 cm to smaller ones.

**Figure 4:**
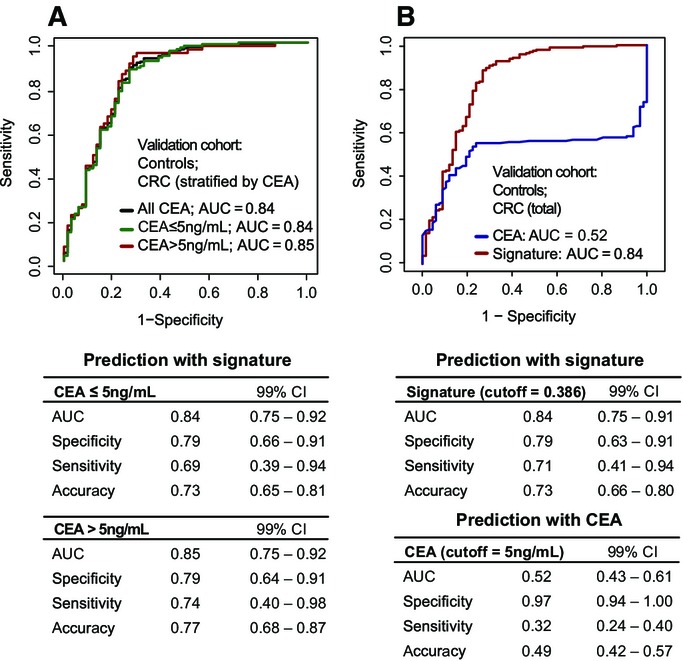
Predictive ability of the protein biomarker signature in the context of CEA in the validation cohort Prediction by the protein biomarker signature stratified by tumor plasma marker CEA cutoff. The subjects with CRC were partitioned according to CEA measurement: positive (CEA > 5 ng/ml, *n* = 62) or negative (CEA ≤ 5 ng/ml, *n* = 130). Each group was discriminated separately against the controls (*n* = 67) using the protein biomarker signature. A threshold of 0.386 was used to read specificity, sensitivity, and accuracy.

Finally, we evaluated the predictive ability of the protein biomarker signature with respect to the tumor plasma marker carcinoembryonic antigen (CEA). In many countries, CEA is measured preoperatively to assist with staging and surgical planning, and also postoperatively at multiple time points to monitor disease recurrence or the response of metastatic disease to systemic therapy (Locker *et al*, 2006). First, we examined the performance of the protein biomarker signature on stratified groups by CEA cutoff to determine whether we can equally detect subjects with very low concentrations of CEA with our signature. We employed the protein biomarker signature for subjects with negative (≤ 5 ng/ml, 68% patients) or positive (> 5 ng/ml, 32% patients) CEA measured at diagnosis in the validation cohort. The areas under the ROC curves for both groups were similar (Fig4A), demonstrating a disease detection independent of patients’ CEA levels.

CRC and control subjects with CEA measurements were classified either by CEA or by the protein biomarker signature.

Second, we assessed the performance of CEA as compared to the protein biomarker signature for diagnosing CRC. Employing the clinical cutoff, as above, 32% of subjects with CRC were detected by CEA alone in the validation cohort (Fig4B). The overall accuracy was 49%, which was much lower than the 72% accuracy of the protein biomarker signature presented here. To further investigate whether CEA had any added impact on CRC detection beyond the protein signature, a new model including CEA and the protein biomarker signature was generated within 10-fold cross-validation on the validation cohort. In accordance with the comparisons above, the performance obtained for the combination of CEA with the signature as compared to the signature alone showed that there is no added benefit of this combination (AUC_CEA+SIGN_ = 0.88 versus AUC_SIGN_ = 0.87, *P* = 0.14, Appendix Fig S8).

## Discussion

The present study proposes a multi-protein biomarker signature for CRC diagnosis. We used an innovative strategy to characterize proteins that play a role in or are regulated as a result of CRC, screen for them in the circulation, and select a subset of proteins with a high predictive ability. To identify disease-associated proteins, we profiled CRC-driven perturbations reflected in the proteome of the tumor epithelia. We acknowledged that tumor-derived proteins in the bloodstream preferentially belong to the class of proteins that are secreted, transported, or enzymatically released into the circulation. We therefore selectively focused on the subproteome of *N-*glycosylated proteins that are destined to reach the extracellular environment. This approach proved successful at detecting biomarker candidates in the systemic circulation and offered a non-invasive readout of patient CRC alterations. The diagnostic potential of the proteins quantified in plasma was assessed in two independent clinical cohorts and led to the prioritization of five proteins, which as a panel proved more predictive than any single protein in isolation.

CRC is a heterogeneous solid malignancy that exhibits genomic differences between patients (Cancer Genome Atlas, 2012) and where multiple cancer modalities (Hanahan & Weinberg, 2011) contribute to its systemic demonstration that can be monitored in the blood circulation (Surinova *et al*, 2015). Given the complex characteristics of CRC, it is unlikely that a single protein marker would be powerful enough to capture this disease across a large set of patients. The most discriminating protein predictors that capture the different aspects of CRC as compared to healthy and benign controls in the circulation were therefore selected into a multivariate signature.

The proteins comprising the diagnostic signature have been previously linked to cancer and play different functional roles. CP and SERPINA3 both belong to the acute-phase reactant proteins and have been associated with systemic inflammation (Bode *et al*, 2012). TIMP1 is involved in the extracellular matrix remodeling as it is the natural inhibitor of the matrix metalloproteinases (MMPs), a group of peptidases involved in degradation of the extracellular matrix (Hadler-Olsen *et al*, 2013). In addition, it has also been proposed that TIMP1 promotes cell proliferation and possesses anti-apoptotic effects (Li *et al*, 1999). PON1 is a known free radical scavenger belonging to the family of serum paraoxonases, possesses antioxidant activities, and was proposed to play a role in carcinogenesis and metastasis status in CRC (Balci *et al*, 2012). The function of LRG1 has been unknown until recently when Wang *et al* reported that it promotes angiogenesis (Wang *et al*, 2013).

All five proteins have been further associated with CRC, and their abundance trends in the plasma of CRC patients and controls are in accordance with the previous reports (Walker & Gray, 1983; Holten-Andersen *et al*, 2002; Balci *et al*, 2012; Ladd *et al*, 2012; Bujanda *et al*, 2013). Regarding predictive ability for CRC diagnosis, LRG1, PON1, and TIMP1 were previously assessed independently. LRG1 was assayed by ELISA and reported to have an increased fold change and predictive ability for CRC detection in a cohort of 58 subjects with CRC and 58 control subjects, and also in a pre-diagnosis cohort of 32 female subjects with CRC and 32 female control subjects (Ladd *et al*, 2012). The extent of differential abundance of a protein between the groups and its predictive ability were in strong accordance with our results. PON1 was examined in the context of the serum oxidative imbalance association with an increased risk of CRC in a cohort of 40 subjects with CRC and 39 controls. Similar to our results, lower serum PON1 levels were found in CRC patients (Bulbuller *et al*, 2013). TIMP1 was assayed by ELISA in blood samples of 179 CRC patients and 225 neoplasm-free participants and was found to have elevated levels in CRC. Its predictive ability to discriminate between these groups was slightly lower to the one found in our cohort (Tao *et al*, 2012). Interestingly, on top of the five proteins in the signature, the exhaustive search procedure highlighted additional proteins as highly ranked (Appendix Fig S3). These proteins can be regarded as “back-up” proteins in case a future assay for a protein within the diagnostic signature will not fulfill the required analytical criteria.

The reported protein biomarker signature accurately predicts subjects with CRC. We have shown that it can detect disease in a stage-independent manner. Tumor size, on the other hand, proved to play an important role in the accuracy of detection, where subjects with larger tumors were detected more readily. This is in agreement with our hypothesis that larger tumors secrete more biomarker into the circulation. In particular, LRG1 and PON1 showed stronger abundance differences in larger tumors.

In the present study, sensitivity of the protein biomarker signature was addressed extensively on two independent cohorts of CRC and control subjects. The second main characteristic of a protein signature is its specificity for the disease in question. The cancer-associated proteins within our signature contain both tumor-derived and systemic response proteins. SERPINA3 and CP can be upregulated in cancerous or non-cancerous inflammatory conditions. It can only be speculated that the tumor-derived inflammation captured with these two proteins may represent a stable upregulation across CRC patients, and this is why SERPINA3 and CP were prioritized into the signature. The differences in inflammation between CRC and non-cancerous conditions such as inflammatory bowel disease (IBD) are not well understood, and a direct assessment of these and other proteins across large enough respective groups is required to gain further insight into the differences between diseases sharing common modalities. The specificity of the signature for CRC is significantly mediated by TIMP1, PON1, and LRG1—the three proteins previously associated with CRC more directly and based on our observation that the abundance of PON1 and LRG1 in the circulation correlated with the tumor size in these patients, pointing toward tumor-specific markers. TIMP1 was previously reported to yield diagnostic value for CRC detection in large cohorts (Holten-Andersen *et al*, 2002). In this study, TIMP1 levels were also monitored for subjects with IBD and no significant differences in plasma TIMP1 levels between healthy and IBD subjects were detected.

Here we report a multivariate signature capturing the most significant CRC demonstrations in the circulation of a large set of cohorts. The claim that a biomarker is specific for the detection of CRC requires an extensive study of the predictive ability of the signature against various types of disease. The study should be performed on samples where clinical data clearly define each homogeneous patient subgroup. The study should employ statistical analysis of the acquired data that is different from the present work, in that it would not only differentiate between CRC and controls, but also show the absence of the signal in other disease subgroups. Since this work utilized a control group that represents the general population of the subjects at risk, and without any additional comorbidities, we cannot claim that the proposed protein biomarker signature is exclusively specific to CRC. We can only hypothesize about the specificity of the signature proteins based on previous reports of their biological roles.

Further development of the protein biomarker signature can lead to a fit-for-purpose clinical grade assay (Carr *et al*, 2014) and to an improvement of its current accuracy. This can be accomplished on both technological and clinical levels. On the measurement level, to reach a high accuracy of CRC detection, further work is needed to improve the assay’s characteristics by: (i) employing a 5-plex SRM assay method with longer acquisition times than the ones used in the 88-plex method to enhance signal-to-noise ratios; (ii) performing absolute quantification with precisely quantified internal standards for the five proteins; and possibly also by (iii) using orthogonal measurements with ELISAs for the signature proteins. On the clinical level, one option is to identify homogeneous subpopulations of subjects where the biomarker signature is particularly effective. This would require studies of a larger size and with more clinical characteristics. Another option is to combine the signature with an existing (e.g. FOBT) or new biomarker to obtain more accurate discrimination from the combination. Finally, performing serial measurements to monitor signature proteins over time, for example, annually or biannually, as is often done for FOBT, is likely to enhance its accuracy.

The current clinical procedures for CRC diagnosis can certainly be improved. Regarding screening, the most commonly employed non-invasive test is the FOBT. Although the test generally shows good specificity, the traditional guaiac-based test does not detect CRC effectively and the detection is particularly poor for early-stage cancers (Booth, 2007; Bretthauer, 2011). Although higher sensitivity and compliance can be achieved with immunochemical fecal occult blood test (Kapidzic *et al*, 2014), a non-invasive blood-based test could represent a more convenient test and thereby enhance compliance further. Further clinical evaluation is necessary to directly compare the performance of our signature with FOBT and other candidate screening tests on the same cohort to assess their relative predictive abilities and to explore a benefit of their combination. Ideally, such evaluation should be performed in a true screening setting, for example, among participants of screening colonoscopy.

## Materials and Methods

### Clinical cohorts

Three independent cohorts were used in this study. The first cohort was designed to be shared between the discovery and the screening phase to increase the likelihood of candidates identified in the tissue and their subsequent detection in the circulation of the same subjects. The subjects were recruited for the purpose of this study at the University Hospital Olomouc in the Czech Republic and were included consecutively as diagnosed. For the validation phase, two large independent cohorts, that is, training and validation cohorts, were designed. The training cohort was comprised of two groups of equal size that include subjects with colorectal cancer (CRC) and subjects representing a control population at risk. Subjects were selected from two ongoing German clinical studies, that is, a prospective screening study (BLiTz) (Hundt *et al*, 2009; Brenner *et al*, 2010) and a case–control study examining the role of colonoscopy in CRC prevention (DACHS+) (Brenner *et al*, 2006, 2007), and their status was colonoscopy-confirmed. The validation cohort included subjects selected consecutively at the University Hospital Olomouc. The control group was comprised of clinically healthy blood donors and subjects with various non-malignant gastrointestinal tract (GIT) conditions such as adenoma, benign condition, diverticular disease, dysplastic polyps, and Crohn’s disease. The CRC group was designed to contain approximately equally sized subgroups of clinical stages. For the validation purpose, tumor diameter was reported for CRC subjects and CEA concentration at diagnosis for all subjects. This study was approved by the ethics committees of the Medical Faculty at the University of Heidelberg, of the Medical Chambers of Baden-Württemberg and Rhineland-Palatinate, and of the University Hospital Olomouc and Faculty of Medicine and Dentistry, Palacky University, Olomouc. Written informed consent was obtained from each participant.

### Collection and preparation of tissue epithelia

Tumor and adjacent normal mucosa tissues were surgically resected based on standard oncological procedures. Frozen tissue was further processed in a pre-cooled cryostat (−20°C). Sections of 7 μm were fixed with 4% formalin and stained with hematoxylin–eosin, and adjacent 40-μm sections were manually dissected and placed in lysis buffer (50% PBS liquid, pH 7.4 (GIBCO, Invitrogen) and 50% 2,2,2-trifluoroethanol (Fluka, 99.9% purity)) until 50 mg of dissected epithelium was obtained per sample.

### Protein extraction and peptide isolation

Tissue epithelia were homogenized in a Microdismembrator S (Sartorius), subjected to protein extraction in lysis buffer (as above), and solubilized with 1% Rapigest (Waters) in 250 mM ammonium bicarbonate. Ultra sonication in a vial-tweeter ultrasonicator (Hielscher) at 4°C was used to further disintegrate the homogenized tissue. Proteins were denatured at 60°C for 2 h, reduced with 5 mM dithiothreitol (DTT) at 60°C for 30 min, and alkylated with 25 mM iodoacetamide (IAA) at 25°C for 45 min in the dark. Samples were diluted to 15% TFE in 100 mM ammonium bicarbonate and proteolyzed with sequencing grade porcine trypsin (Promega) at a protease to substrate ratio of 1:100 at 37°C for 15 h. Peptide mixtures were desalted with Sep-Pak tC18 cartridges (Waters, Milford, MA, USA), eluted with 50% acetonitrile/0.1% formic acid, evaporated to dryness, and resolubilized in 100 μl 20 mM sodium acetate and 100 mM sodium chloride, pH 5.

### Glycopeptide enrichment

Glycopeptides were isolated as described previously (Zhang *et al*, 2003). *N*-linked glycosylated peptides were released with *N*-glycosidase F (PNGase F; Roche and New England Biolabs). Formerly glycosylated peptides were desalted as above and resolubilized in 100 μl HPLC grade water/2% acetonitrile/0.1% formic acid.

### Discovery-driven LC-MS of tissue *N*-glycosites

LC-MS/MS analyses were carried out on a hybrid LTQ-FT-ICR mass spectrometer (Thermo Electron) interfaced to a nanoelectrospray ion source (Thermo Electron) coupled to a Tempo NanoLC system (ABI/MDS Sciex). Two microliters of *N*-glycosite samples was loaded from a cooled (4°C) autosampler (ABI/MDS Sciex) and separated on a 15-cm fused silica emitter, 75 μm diameter, packed in-house with Magic C18 AQ 3 μm resin (Michrom BioResources) using a linear gradient from 5 to 35% acetonitrile/0.1% formic acid over 60 (for samples from patients 1, 3, 5, 6, and 7) or 90 min (for samples from patients 8, 10–14, 16, 17, 19, 22, and 24), at a flow rate of 300 nl/min. In data-dependent analysis (DDA) mode, each MS1 survey scan acquired in the ICR cell with an overall cycle time of approximately 1 s, exceeding 150 counts, was followed by collision-induced dissociation (CID) acquired in the LTQ of the three most abundant precursor ions with a dynamic exclusion of 30 s. For MS1, 10^6^ ions were accumulated in the ICR cell over a maximum time of 500 ms and scanned at a resolution of 100,000 full-width at half-maximum nominal resolution settings. MS2 spectra were acquired using the normal scan mode, a target setting of 10^4^ ions, and an accumulation time of maximally 250 ms. Charge state screening was used to select ions with at least two charges and to reject ions with unassigned charge state. Normalized collision energy was set to 32%, and one microscan was acquired for each spectrum. Samples were acquired in triplicates (for samples from patients 1, 3, 5, 6, and 7) or duplicates (for samples from patients 8, 10–14, 16, 17, 19, 22, and 24).

### Protein identification and CRC *N*-glycosite PeptideAtlas compilation

Raw data files were centroided and converted to the mzXML format with ReAdW (http://tools.proteomecenter.org/wiki/index.php?title=Software:ReAdW). MS/MS spectra were searched using the SORCERER-SEQUEST search tool against a semitryptic human UniProt protein database downloaded on 12 October 2010 (http://www.uniprot.org). The search criteria were set to cleavage after lysine or arginine, unless followed by proline; at least at one tryptic terminus; maximally one missed cleavage allowed; cysteine carbamidomethylation set as fixed modification; methionine oxidation and asparagine deamidation set as variable modifications; monoisotopic parent and fragment ion masses; and precursor ion mass tolerance of 50 ppm. The database search results were further validated with the Trans-Proteomic Pipeline (TPP), with a false positive rate set to 1% on both the peptide and protein level, as determined by PeptideProphet (Keller *et al*, 2002) and ProteinProphet (Nesvizhskii *et al*, 2003), respectively. Data and search results were uploaded to the PeptideAtlas and can be accessed at https://db.systemsbiology.net/sbeams/cgi/PeptideAtlas/buildDetails?atlas_build_id=374.

### Protein topology prediction

Prediction of secondary protein structure was performed from the amino acid sequence with Phobius (http://phobius.sbc.su.se/).

### Relative quantification and statistical testing of CRC tissue *N*-glycosites

Peptides were filtered for the glycosylation motif. Raw data were converted to a profile mzXML format as above. Label-free quantification of all 74 runs together with their search results was performed by OpenMS 1.7 (Sturm *et al*, 2008) as described elsewhere (Weisser *et al*, 2013). Quantitative features annotated with peptide sequences were exported, the abundance values were log_2_-transformed, and a scale-normalization procedure (Smyth & Speed, 2003) was applied. Features missing in more than five-sixths of the runs or in an entire experimental group were removed from the dataset. Protein quantification was performed with MSstats (v1.0) (Choi *et al*, 2014). Comparisons of mean protein abundance between conditions were carried out with MSstats with restricted scope of conclusions for biological replication, and *P*-values were adjusted to control for the false discovery rate using the Benjamini–Hochberg procedure (Benjamini & Hochberg, 1995).

### Blood collection and plasma preparation

Blood was drawn prior to surgery from the cubital vein and collected into tubes processed with EDTA. In the discovery and validation cohort, blood was directly centrifuged at 6,067× *g* for 3 min at 4°C. Plasma was collected into a new tube, frozen at −20°C, and stored at −80°C. In the training cohort, blood was drawn before bowel preparation for colonoscopy or prior to large bowel surgery and centrifuged at 2,123× *g* for 10 min.

### Glycoprotein enrichment from plasma

Glycoproteins were isolated as described previously (Zhang *et al*, 2003) and above, starting with 50 μl of plasma. Prior to the enrichment, bovine standard *N*-glycoproteins (fetuin and alpha-1-acid glycoprotein) were spiked into samples at equal concentration (10 pmol/protein) to control experimental variation. Counter to above, glycoproteins were first oxidized, immobilized on resin; non-bound proteins were thoroughly washed away with urea buffer (8 M urea, 100 mM ammonium bicarbonate, 0.1% SDS, 5 mM EDTA) and then proteolyzed at 2 M urea, and *N*-linked glycosylated peptides were enzymatically released as above. The protocol was adapted to a Sirocco 96-well plate (Waters) where Affi-gel hydrazine resin (Bio-Rad) was used. Formerly glycosylated peptides were desalted as above in 96-well MacroSpin column plates filled with Vydac C18 silica (The Nest Group Inc.) and resolubilized in 100 μl HPLC grade water/2% acetonitrile/0.1% formic acid. Special focus was given to sample processing of the training and validation cohorts. Samples were block-randomized according to their clinical features and processed in a blinded way to prevent the introduction of experimental bias.

### Targeted LC-SRM analysis of plasma *N*-glycosites

Samples from the screening and validation cohorts were analyzed on a hybrid triple quadrupole/ion trap (4000 QTrap, ABI/MDS Sciex) equipped with a nanoelectrospray ion source and a Tempo NanoLC system (ABI/MDS Sciex) coupled to a 15-cm fused silica emitter, 75 μm diameter, packed in-house with a Magic C18 AQ 5 μm resin (Michrom BioResources). Samples were loaded from a cooled (4°C) autosampler (ABI/MDS Sciex) and separated over a linear gradient from 5 to 35% acetonitrile/0.1% formic acid over 35 min, at a flow rate of 300 nl/min. The instrument was operated in scheduled SRM mode (retention time window of 300 s, target scan time of 3 s), at a unit resolution (0.7 *m/z* half-maximum peak width) of both Q1 and Q3 analyzers. Samples from the training cohort were analyzed on TSQ Vantage (Thermo Fischer Scientific) equipped with a nanoelectrospray ion source. Chromatographic separation of peptides was carried out on a nano-LC system (Eksigent). In each injection, peptides were loaded onto a 75-μm diameter and 10.5-cm-long fused silica microcapillary reverse-phase column, in-house packed with Magic C18 AQ material (200 Å pore, 5 μm diameter; Michrom BioResources). For peptide separation, a linear 40-min gradient from 2 to 40% solvent B (solvent A: 98% water, 2% acetonitrile, 0.1% formic acid; solvent B: 98% acetonitrile, 2% water, 0.1% formic acid) at a 300 nl/min flow rate was applied. The mass spectrometer was operated in the positive ion mode using ESI with a capillary temperature of 270°C, a spray voltage of +1,350 V, and a collision gas pressure of 1.5 mTorr. SRM transitions were monitored with a mass window of 0.7 half-maximum peak width (unit resolution) in Q1 and Q3. All of the measurements were performed in scheduled mode, applying a retention time window of 3 min and a cycle time of 2 s. SRM assays were retrieved from the *N*-glycosite SRM atlas (http://www.srmatlas.org/) (Bujanda *et al*, 2013), reanalyzed to select the best transitions for endogenous detection in plasma, split to multiple SRM methods, or used to optimize a single SRM method. Internal standard peptides labeled with heavy isotopes at the C-terminal lysine or arginine, +8 or +10 Da, respectively (Thermo Scientific, Sigma-Aldrich, or JPT Peptide Technology), were used to validate peptide identity by analogy of chromatographic and fragmentation properties to the reference. Similar to above, efforts were made to prevent bias during measurements of the training and validation cohorts. A data collection sequence was generated by block randomization of experimental groups, and data were acquired in a blinded way. Raw data and SRM transition files can be accessed, queried, and downloaded via PASSEL (Farrah *et al*, 2012) of the SRMAtlas by selecting the respective datasets from the drop-down menu of SRM experiments (https://db.systemsbiology.net/sbeams/cgi/PeptideAtlas/GetSELTransitions?SBEAMSentrycode=Crcpass2013).

### Relative quantification and statistical analysis of plasma *N*-glycosites

Raw data files from the screening and validation cohorts were uploaded to MultiQuant 1.2 (Applied Biosystems) and files from the training cohort to Skyline (MacLean *et al*, 2010) to perform automatic SRM peak integration. Normalization was applied to logarithm base 2-transformed peak areas, separately for each cohort. The normalization relied on: (i) internal stable isotope-labeled standard reference peptides for each targeted endogenous peptide, to account for systematic shifts in the signal during data acquisition, and (ii) internal standard bovine proteins across runs, to account for batch effects and other potential artifacts that could have occurred prior to data acquisition. First, protein-level constant normalization was applied, which equalized the median reference intensities for each protein across all runs and shifted all the endogenous intensities in a run by the same amount. Second, the individual peptide intensities of standard proteins were modeled to obtain a single summary value that quantifies the abundance of the proteins in each sample on a relative scale. These quantities were correlated with the median of the total intensities of plasma samples by Pearson’s correlation. Bovine fetuin had a correlation of > 0.6, was considered stable, and was used for data normalization in both cohorts. All the endogenous plasma intensities in a run were shifted by the sample value constant to make the median of sample representative intensities of the standard protein across all runs equal in order to remove the systematic bias created during sample preparation. Comparisons of mean protein abundance between groups were carried out with MSstats (v2.3.5) (Choi *et al*, 2014) at expanded scope of conclusions for technical replication and at restricted scope of conclusions for biological replication. *P*-values were adjusted as above. Normalized data were also used to estimate model-based estimation of sample quantification for individual proteins. Specifically, the relative protein abundances were summarized across all peptide intensities for each subject using MSstats.

### Predictive analysis

To make the intensities comparable for the purpose of predictive analysis, the median normalized log_2_-relative quantifications of the validation cohort were equalized with the median normalized log_2_-relative quantifications of the training cohort. Missing relative quantifications were imputed for a given protein with a minimal relative quantification observed for that protein across all runs in the same cohort, representing its limit of detection. Unsupervised hierarchical clustering with Euclidean distance and Ward linkage was employed to cluster samples by similarity of normalized relative protein abundance, and visualized in a heatmap. For prediction analysis, proteins for which more than 40% of the subjects had missing relative quantifications were filtered out. Tenfold cross-validation was used to find the protein biomarker signature in the training cohort. For each fold, proteins were tested for differential abundance between CRC and controls using the nine-tenths of the subjects (FDR < 0.05 and fold change cutoff ± 1.1). The relative abundances of the proteins with significant tests were used as input to logistic regression. The subset of proteins was further reduced by stepwise selection [i.e. by repetitively adding or dropping proteins until minimizing the Akaike information criterion (AIC)]. The predictive accuracy of the selected model was evaluated on the remaining one-tenth of the subjects in the training cohort and summarized with an ROC curve. The process was repeated ten times, by systematically rotating the one-tenth of the left-out subjects. The final predictive model was comprised of proteins which were selected more than five times among the tenfolds. The performance of the final model was assessed on the validation dataset. The threshold was determined based on the best accuracy in the training dataset. To evaluate the stability of the final model, the procedure was repeated an additional three times on differently partitioned subjects within 10-fold cross-validation. Moreover, eightfold cross-validation was also employed. Identical methodology to the 10-fold procedure was applied, except that the final consensus model comprised proteins selected more than four times among the eightfolds. The pROC package in R was used to draw ROC curves, calculate areas under the curves, and perform the inference. Inference for the AUCs was done using bootstrap with B = 2,000 bootstrap samples.

### Exhaustive search of protein predictors

All possible combinations of one to five proteins were systematically collected by brute force search to form logistic regression models. Every logistic regression model was validated on the training dataset with 100-fold bootstrapped cross-validation (Efron & Tibshirani, 1993). Validated models were ranked according to their median AUC. Proteins in a set of high-performing models that have an identical cross-validation performance were ranked according to their frequency among models.

## For more information

UniProt, the Universal Protein Resource for protein sequence and annotation data. http://www.uniprot.org/

SRMAtlas, a compendium of targeted proteomics assays for the detection and quantification of proteins in complex proteome digests by mass spectrometry.

http://www.srmatlas.org/

PASSEL, the PeptideAtlas SRM Experiment Library for the submission, dissemination, and reuse of SRM experimental data. http://www.peptideatlas.org/passel/

MSstats, a statistical tool for quantitative mass spectrometry-based proteomics employing a family of linear mixed-effects models. http://www.msstats.org/

## Supporting Information

**Appendix**

**Table EV1**

**Table EV2**

**Table EV3**

**Review Process File**

## References

[b1] Agresti A (2012). Categorical Data Analysis.

[b2] Anderson NL, Anderson NG (2002). The human plasma proteome: history, character, and diagnostic prospects. Mol Cell Proteomics.

[b3] Balci H, Genc H, Papila C, Can G, Papila B, Yanardag H, Uzun H (2012). Serum lipid hydroperoxide levels and paraoxonase activity in patients with lung, breast, and colorectal cancer. J Clin Lab Anal.

[b4] Benjamini Y, Hochberg Y (1995). Controlling the false discovery rate – a practical and powerful approach to multiple testing. J Roy Stat Soc B Met.

[b5] Bock T, Bausch-Fluck D, Hofmann A, Wollscheid B (2012). CD proteome and beyond – technologies for targeting the immune cell surfaceome. Front Biosci.

[b6] Bode JG, Albrecht U, Haussinger D, Heinrich PC, Schaper F (2012). Hepatic acute phase proteins–regulation by IL-6- and IL-1-type cytokines involving STAT3 and its crosstalk with NF-kappaB-dependent signaling. Eur J Cell Biol.

[b7] Booth RA (2007). Minimally invasive biomarkers for detection and staging of colorectal cancer. Cancer Lett.

[b8] Brenner H, Chang-Claude J, Seiler CM, Sturmer T, Hoffmeister M (2006). Does a negative screening colonoscopy ever need to be repeated?. Gut.

[b9] Brenner H, Chang-Claude J, Seiler CM, Sturmer T, Hoffmeister M (2007). Case-control study supports extension of surveillance interval after colonoscopic polypectomy to at least 5 year. Am J Gastroenterol.

[b10] Brenner H, Haug U, Hundt S (2010). Inter-test agreement and quantitative cross-validation of immunochromatographical fecal occult blood tests. Int J Cancer.

[b11] Bretthauer M (2011). Colorectal cancer screening. J Intern Med.

[b12] Bujanda L, Sarasqueta C, Cosme A, Hijona E, Enriquez-Navascues JM, Placer C, Villarreal E, Herreros-Villanueva M, Giraldez MD, Gironella M (2013). Evaluation of alpha 1-antitrypsin and the levels of mRNA expression of matrix metalloproteinase 7, urokinase type plasminogen activator receptor and COX-2 for the diagnosis of colorectal cancer. PLoS ONE.

[b13] Bulbuller N, Eren E, Ellidag HY, Oner OZ, Sezer C, Aydin O, Yilmaz N (2013). Diagnostic value of thiols, paraoxonase 1, arylesterase and oxidative balance in colorectal cancer in human. Neoplasma.

[b14] Cancer Genome Atlas N (2012). Comprehensive molecular characterization of human colon and rectal cancer. Nature.

[b15] Carr SA, Abbatiello SE, Ackermann BL, Borchers C, Domon B, Deutsch EW, Grant RP, Hoofnagle AN, Hüttenhain R, Koomen JM (2014). Targeted peptide measurements in biology and medicine: best practices for mass spectrometry-based assay development using a fit-for-purpose approach. Mol Cell Proteomics.

[b16] Choi M, Chang CY, Clough T, Broudy D, Killeen T, MacLean B, Vitek O (2014). MSstats: an R package for statistical analysis of quantitative mass spectrometry-based proteomic experiments. Bioinformatics.

[b17] Cleveland WS, Grosse E, Shyu MJ, Chambers JM, Hastie T (1992). Local Regression Models. Statistical Models in S.

[b18] Efron B, Tibshirani R (1993). An Introduction to the Bootstrap.

[b19] Farrah T, Deutsch EW, Kreisberg R, Sun Z, Campbell DS, Mendoza L, Kusebauch U, Brusniak MY, Hüttenhain R, Schiess R (2012). PASSEL: the peptideAtlas SRMexperiment library. Proteomics.

[b20] Haab BB, Geierstanger BH, Michailidis G, Vitzthum F, Forrester S, Okon R, Saviranta P, Brinker A, Sorette M, Perlee L (2005). Immunoassay and antibody microarray analysis of the HUPO Plasma Proteome Project reference specimens: systematic variation between sample types and calibration of mass spectrometry data. Proteomics.

[b21] Hadler-Olsen E, Winberg JO, Uhlin-Hansen L (2013). Matrix metalloproteinases in cancer: their value as diagnostic and prognostic markers and therapeutic targets. Tumour Biol.

[b22] Hanahan D, Weinberg RA (2011). Hallmarks of cancer: the next generation. Cell.

[b23] Holten-Andersen MN, Christensen IJ, Nielsen HJ, Stephens RW, Jensen V, Nielsen OH, Sorensen S, Overgaard J, Lilja H, Harris A (2002). Total levels of tissue inhibitor of metalloproteinases 1 in plasma yield high diagnostic sensitivity and specificity in patients with colon cancer. Clin Cancer Res.

[b24] Hortin GL, Sviridov D, Anderson NL (2008). High-abundance polypeptides of the human plasma proteome comprising the top 4 logs of polypeptide abundance. Clin Chem.

[b25] Hundt S, Haug U, Brenner H (2009). Comparative evaluation of immunochemical fecal occult blood tests for colorectal adenoma detection. Ann Intern Med.

[b26] Kapidzic A, Grobbee EJ, Hol L, van Roon AH, van Vuuren AJ, Spijker W, Izelaar K, van Ballegooijen M, Kuipers EJ, van Leerdam ME (2014). Attendance and yield over three rounds of population-based fecal immunochemical test screening. Am J Gastroenterol.

[b27] Keller A, Nesvizhskii AI, Kolker E, Aebersold R (2002). Empirical statistical model to estimate the accuracy of peptide identifications made by MS/MS and database search. Anal Chem.

[b28] Ladd JJ, Busald T, Johnson MM, Zhang Q, Pitteri SJ, Wang H, Brenner DE, Lampe PD, Kucherlapati R, Feng Z (2012). Increased plasma levels of the APC-interacting protein MAPRE1, LRG1, and IGFBP2 preceding a diagnosis of colorectal cancer in women. Cancer Prev Res.

[b29] Li G, Fridman R, Kim HR (1999). Tissue inhibitor of metalloproteinase-1 inhibits apoptosis of human breast epithelial cells. Cancer Res.

[b30] Locker GY, Hamilton S, Harris J, Jessup JM, Kemeny N, Macdonald JS, Somerfield MR, Hayes DF, Bast RC (2006). ASCO 2006 update of recommendations for the use of tumor markers in gastrointestinal cancer. J Clin Oncol.

[b31] MacLean B, Tomazela DM, Shulman N, Chambers M, Finney GL, Frewen B, Kern R, Tabb DL, Liebler DC, MacCoss MJ (2010). Skyline: an open source document editor for creating and analyzing targeted proteomics experiments. Bioinformatics.

[b32] Nesvizhskii AI, Keller A, Kolker E, Aebersold R (2003). A statistical model for identifying proteins by tandem mass spectrometry. Anal Chem.

[b33] Polanski M, Anderson NL (2007). A list of candidate cancer biomarkers for targeted proteomics. Biomark Insights.

[b34] Roth J (2002). Protein N-glycosylation along the secretory pathway: relationship to organelle topography and function, protein quality control, and cell interactions. Chem Rev.

[b35] Schiess R, Wollscheid B, Aebersold R (2009). Targeted proteomic strategy for clinical biomarker discovery. Mol Oncol.

[b36] Smyth GK, Speed T (2003). Normalization of cDNA microarray data. Methods.

[b37] Sobin LH (2003). TNM: evolution and relation to other prognostic factors. Semin Surg Oncol.

[b38] Sturm M, Bertsch A, Gropl C, Hildebrandt A, Hussong R, Lange E, Pfeifer N, Schulz-Trieglaff O, Zerck A, Reinert K (2008). OpenMS – an open-source software framework for mass spectrometry. BMC Bioinformatics.

[b39] Surinova S, Radová L, Choi M, Srovnal J, Brenner H, Vitek O, Hajdúch M, Aebersold R (2015). Non-invasive prognostic protein biomarker signatures associated with colorectal cancer. EMBO Mol Med.

[b40] Tao S, Haug U, Kuhn K, Brenner H (2012). Comparison and combination of blood-based inflammatory markers with faecal occult blood tests for non-invasive colorectal cancer screening. Br J Cancer.

[b41] Walker C, Gray BN (1983). Acute-phase reactant proteins and carcinoembryonic antigen in cancer of the colon and rectum. Cancer.

[b42] Wang X, Abraham S, McKenzie JA, Jeffs N, Swire M, Tripathi VB, Luhmann UF, Lange CA, Zhai Z, Arthur HM (2013). LRG1 promotes angiogenesis by modulating endothelial TGF-beta signalling. Nature.

[b43] Weisser H, Nahnsen S, Grossmann J, Nilse L, Quandt A, Brauer H, Sturm M, Kenar E, Kohlbacher O, Aebersold R (2013). An automated pipeline for high-throughput label-free quantitative proteomics. J Proteome Res.

[b44] Zhang H, Li XJ, Martin DB, Aebersold R (2003). Identification and quantification of N-linked glycoproteins using hydrazide chemistry, stable isotope labeling and mass spectrometry. Nat Biotechnol.

[b45] Zhang H, Liu AY, Loriaux P, Wollscheid B, Zhou Y, Watts JD, Aebersold R (2007). Mass spectrometric detection of tissue proteins in plasma. Mol Cell Proteomics.

