# Supplementary material for: Prediction of colorectal cancer diagnosis based on circulating plasma proteins
Source: EMBO Mol Med. 2015 Aug 7;7(9):1166–78. doi: 10.15252/emmm.201404873 (PMC4568950; doi:10.15252/emmm.201404873)
Supplement: Supplementary file 1 [file emmm0007-1166-sd1.pdf]

Appendix

Prediction of colorectal cancer diagnosis based on circulating plasma proteins

Silvia Surinova, Meena Choi, Sha Tao, Peter Schüffler, Ching-Yun Chang, Tim Clough, Kamil Vysloužil, Marta Dziechciarková, Josef Srovnal, Yansheng Liu, Mariette Matondo, Ruth Hüttenhain, Hendrik Weisser, Joachim Buhmann, Marián Hajdúch, Hermann Brenner, Olga Vitek, Ruedi Aebersold

Table of contents

Appendix methods

Transformation of fold changes and standard errors from the log scale to the original scale

Appendix figures

Appendix Fig. S1. Preparation of tissue epithelia for proteomic analysis

Appendix Fig. S2. Multivariate logistic regression model used to evaluate the predictive ability of the biomarker signature

Appendix Fig. S3. Exhaustive search for all predictor models

Appendix Fig. S4. The relative intensities of the signature proteins

Appendix Fig. S5. Specificity evaluation of the control group in cohort 1

Appendix Fig. S6. The predictive ability of individual signature proteins

Appendix Fig. S7. Evaluation of the signature's predictive ability with or without age in the training and validation cohorts

Appendix Fig. S8. Comparative assessment of predictive ability of the protein biomarker signature and CEA in the validation cohort by cross validation

Appendix tables

Appendix Table S1. Patient cohort used for the discovery and screening of biomarker candidates

Appendix Table S2. Biomarker signature development within 10-fold cross validation (CV)

Appendix Table S3. Reproducibility assessment of biomarker signature development within 10- and 8-fold CV repeated three times

Appendix methods

Transformation of fold changes and standard errors from the log scale to the original scale

By Delta method,

if the sequence of random variable, X_n satisfies,

$$\sqrt{n}[X_n - \theta] \xrightarrow{D} \mathcal{N}(0, \sigma^2)$$

where θ and σ^2 are finite valued constants then,

$$\sqrt{n}[g(X_n) - g(\theta)] \xrightarrow{D} \mathcal{N}(0, \sigma^2[g'(\theta)]^2)$$

in our case,

$$X_n = log_2(Y_n)$$

where, Y_n is original scale fold change, and X_n is log 2 transformed fold change. Then,

$$Y_n = g(X_n) = 2^{X_n}$$

$$g(\theta) = 2^\theta$$
$$g'(\theta) = (2^\theta)' = ln(2)2^\theta$$

Therefore, FC with original scale $= 2^{\text{log2 FC}}$, and SE with original scale = SE log2 scale $\times$ $ln(2)2^{\text{log2 FC}}$.

Training	log2 FC	SE for log2	FC	SE for original
CP	0.38098	0.0507	1.3022	0.04577
PON1	-0.1304	0.0617	0.91359	0.03908
SERPINA3	0.3592	0.06251	1.2827	0.05558
LRG1	0.3066	0.03368	1.2368	0.02887
TIMP1	0.2503	0.03178	1.1894	0.0262

Validation	log2 FC	SE for log2	FC	SE for original
CP	0.70048	0.03015	1.6251	0.03396
PON1	-0.272	0.05618	0.8282	0.03225
SERPINA3	0.2855	0.04792	1.2188	0.04048
LRG1	0.5974	0.03445	1.513016	0.036134
TIMP1	0.5188795	0.042727	1.4328	0.042435

Appendix figures

Appendix Fig. S1. Preparation of tissue epithelia for proteomic analysis. Formalin-fixed 7µm sections were stained with Hematoxilin-Eosin to determine tissue orientation, and adjacent 40µm sections were manually dissected and compiled under denaturing conditions. Left and right image was taken prior and during dissection, respectively.

Appendix Fig. S2. Multivariate logistic regression model used to evaluate the predictive ability of the biomarker signature. The parameters of the logistic regression model and the standard errors of these parameters based on the logistic regression model fit are reported.

Appendix Fig. S3. Exhaustive search for all predictor models. All hypothetical 1-5 protein logistic regression models were collected brute force search and validated by 100-fold bootstrap cross-validation. Proteins were ranked by their median AUROC. The protein occurrence in models

represents proteins most frequently selected into the most 2097 high performing predictor models. Proteins in bold present the proteins from the diagnostic protein signature.

Appendix Fig. S4. The relative intensities of the signature proteins. The log2 intensities have been estimated from the linear model. Boxplots of the signature proteins significant between the groups of CRC and control subjects were plotted for the a, training, and b, validation data set.

Appendix Fig. S5. Specificity evaluation of the control group in cohort 1. a, The training data set was partitioned into five folds and the protein biomarker signature was used to classify all the subjects of the training cohort and also specific control subsets in detail. The subject with hyperplastic polyps and non-advanced adenomas were grouped into a group of subjects with ‘pre-lesions’. Subjects with a negative colonoscopy test were assigned to the ‘no lesion’ group. The specificity of prediction was evaluated and fold 2 (in bold) represents the pseudomedian fold of the cross validation procedure. b, An additional cohort of subjects with advanced adenomas (n=50) was used as a new validation cohort of control subjects. The protein biomarker signature was used to classify these subjects.

Appendix Fig. S6. The predictive ability of individual signature proteins. Areas under the ROC curves were obtained from predictors of individual proteins on the validation data set.

Appendix Fig. S7. Evaluation of the signature’s predictive ability with or without age in the training and validation cohorts. Using the original data subsets, proteins were selected into predictive models with or without age within 10-fold cross validation. Proteins selected in at least five folds are listed. The parameters of the model with age are reported. The difference in areas under the ROC curves resulting from the two model predictions is assessed.

Appendix Fig. S8. Comparative assessment of predictive ability of the protein biomarker signature and CEA in the validation cohort by cross validation. CRC and control subjects with CEA measurements (n=192) were classified with CEA, the protein biomarker signature, or a combination of CEA + signature. a, The cross-validated performance within 10 folds is reported for each of the predictors. b, The pseudomedian areas under the ROC curve for each of the cross-validated predictions and the areas between the 25th and 75th percentile are plotted. The mean differences in AUC values were statistically tested by a paired t-test and the p-values were assessed by 2000 bootstrap repetitions.

Appendix tables

Appendix Table S1. Patient cohort used for the discovery and screening of biomarker candidates. Patients are grouped according to clinical stage into a disease progression group (early or advanced) and a disease localization group (localized or metastatic). Patients used in the discovery phase (1) and/or screening phase (2) of the study are indicated. Age represents subject’s age at diagnosis.

Patient #	Gender	Age	Progression	Localization	Stage	Study phase
1	M	61	Advanced	Metastatic	IV	1
2	M	66	Advanced	Localized	II	2
3	M	68	Advanced	Localized	II	1
4	M	82	Advanced	Localized	III	2
5	M	58	Advanced	Localized	III	1+2
6	M	65	Advanced	Localized	II	1+2
7	M	79	Advanced	Metastatic	IV	1
8	F	66	Early	Localized	I	1+2
9	M	70	Advanced	Localized	II	2
10	F	47	Early	Localized	I	1+2
11	M	73	Early	Localized	I	1+2
12	M	70	Early	Localized	I	1+2
13	M	55	Early	Localized	I	1+2
14	M	56	Early	Localized	I	1+2
15	F	78	Advanced	Metastatic	IV	2
16	M	57	Early	Localized	I	1+2
17	M	79	Advanced	Localized	III	1+2
18	M	72	Early	Localized	I	2
19	M	57	Early	Localized	I	1+2
20	F	52	Early	Localized	I	2
21	F	72	Early	Localized	I	-
22	F	53	Early	Localized	I	1+2
23	F	51	Advanced	Metastatic	IV	-
24	M	54	Early	Localized	I	1+2

Appendix Table S2. Biomarker signature development within 10-fold cross validation (CV). **a**, Differentially abundant proteins characterized as significant in the individual folds of the training dataset. **b**, Proteins selected into logistic regression models in individual folds. The consensus model contains proteins with a high frequency of occurrence in the individual folds. AUC values are reported.

**a**	**Significant proteins for each fold (FDR<0.05, fold change cut-off ±1.1)**
Fold	Differentially abundant proteins
1	A1AG2,CP,CTSD,ECM1,FHR3,HP,ITIH4,LGALS3BP,LRG1,MMRN1,ORM1,PON1,SERPINA1,SERPINA3,THBS1,TIMP1,CD44,CFH
2	A1AG2,CD44,CFH,CP,CTSD,ECM1,FHR3,HP,ITIH4,LGALS3BP,LRG1,MMRN1,ORM1,SERPINA1,SERPINA3,TIMP1,SERPINA7,F5
3	A1AG2,CD44,CFH,CP,CTSD,ECM1,FGG,FHR3,HP,ITIH4,LGALS3BP,LRG1,MMRN1,ORM1,SERPINA1,SERPINA3,TIMP1,VTN
4	A1AG2,CFH,CP,CTSD,ECM1,FHR3,ITIH4,LGALS3BP,LRG1,MMRN1,ORM1,SERPINA1,SERPINA3,TIMP1,HP,CD44,PRG4
5	A1AG2,CP,CTSD,ECM1,FHR3,FN1,ITIH4,LGALS3BP,LRG1,MMRN1,ORM1,PON1,SERPINA1,SERPINA3,TIMP1,HP,CD44,PRG4
6	A1AG2,CFH,CP,CTSD,ECM1,FHR3,FN1,HP,IGHG2,ITIH4,LGALS3BP,LRG1,MMRN1,ORM1,PON1,SERPINA1,SERPINA3,TIMP1,CD44,FCGBP
7	A1AG2,CP,CTSD,ECM1,FHR3,IGHG2,ITIH4,LGALS3BP,LRG1,MMRN1,ORM1,PON1,SERPINA1,SERPINA3,TIMP1,CFH,CD44
8	A1AG2,CFH,CP,CTSD,ECM1,FHR3,ITIH4,LGALS3BP,LRG1,MMRN1,ORM1,PON1,SERPINA1,SERPINA3,TIMP1,CD44,F5,HP
9	A1AG2,CP,CTSD,ECM1,FHR3,FN1,IGHA2,IGHG2,LGALS3BP,LRG1,MMRN1,ORM1,PON1,SER

	PINA1,SERPINA3,TIMP1,CD44,HP,PRG4
10	A1AG2,CFH,CP,CTSD,ECM1,FHR3,FN1,ITIH4,LGALS3BP,LRG1,MMRN1,ORM1,SERPINA1,SERPINA3,TIMP1,HP,CD44

**b**	**Significant proteins selected into logistic regression models by stepwise selection**				
Fold	Predictive logistic regression models	Sub-Training (9/10 dataset 1)	Sub-Validation (1/10 dataset 1)	Training (dataset 1)	Validation (dataset 2)
1	CP+LRG1+PON1+SERPINA3	0.75	0.75	0.75	0.83
2	CD44+CP+ITIH4+LRG1+ORM1	0.73	0.55	0.72	0.82
3	CP+FGG+HP+ITIH4+ORM1+TIMP1	0.73	0.48	0.71	0.82
4	LRG1+MMRN1+ORM1+PRG4	0.75	0.61	0.73	0.80
5	CP+PON1+SERPINA3+TIMP1	0.75	0.73	0.74	0.84
6	CP+CTSD+IGHG2+PON1+SERPINA3	0.76	0.72	0.76	0.84
7	CP+IGHG2+PON1+SERPINA3+TIMP1	0.77	0.68	0.77	0.85
8	CP+ECM1+PON1+SERPINA3	0.75	0.43	0.74	0.81
9	CP+IGHA2+IGHG2+LGALS3BP+PON1+SERPINA3	0.79	0.74	0.77	0.85
10	CP+FHR3+FN1+ITIH4+LRG1+TIMP1	0.75	0.54	0.72	0.84
Consensus	CP+PON1+SERPINA3+LRG1+TIMP1			0.75	0.84

Appendix Table S3. Reproducibility assessment of biomarker signature development within 10- and 8-fold CV repeated three times. AUC values are reported.

**Try 1 10-fold CV**	**Significant proteins selected into logistic regression models by stepwise selection**				
Fold	Predictive logistic regression models	Sub-Training (9/10 dataset 1)	Sub-Validation (1/10 dataset 1)	Training (dataset 1)	Validation (dataset 2)
1	CFH + CP + ECM1 + FN1 + LGALS3BP + LRG1 + ORM1 + PON1 + SERPINA3	0.81	0.65		
2	CP + IGHG2 + PON1 + SERPINA3 + TIMP1	0.77	0.63		
3	CP + ITIH4 + LRG1 + TIMP1	0.72	0.67		
4	CP + IGHG2 + MMRN1 + PON1 + SERPINA3 + PRG4	0.77	0.8		
5	CP + ITIH4 + LRG1 + MMRN1	0.72	0.62		
6	CP + ITIH4 + LRG1 + TIMP1	0.71	0.72		
7	CP + ITIH4 + LRG1	0.71	0.64		
8	CP + ITIH4 + LRG1 + TIMP1	0.73	0.56		
9	CP + ITIH4 + LRG1 + TIMP1	0.70	0.8		
10	CP + IGHA2 + ITIH4 + LRG1 + TIMP1	0.71	0.86		
Consensus	CP+LRG1+ITIH4+TIMP1			0.71	0.83
**Try 2 10-fold CV**	**Significant proteins selected into logistic regression models by stepwise selection**				
Fold	Predictive logistic regression models	Sub-Training (9/10 dataset 1)	Sub-Validation (1/10 dataset 1)	Training (dataset 1)	Validation (dataset 2)
1	CP + PON1 + TIMP1 + SERPINA3 + PRG4	0.75	0.71		
2	LGALS3BP + MMRN1 + ORM1 + PRG4	0.73	0.45		
3	IGHA2 + LGALS3BP + ORM1	0.73	0.64		

	+ TIMP1 + PRG4				
4	CP + IGHG2 + PON1 + SERPINA3	0.77	0.70		
5	CP + ITIH4 + LRG1 + TIMP1	0.72	0.63		
6	CP + ITIH4 + LRG1	0.72	0.63		
7	A1AG2 + CP + ITIH4 + ORM1 + SERPINA3 + TIMP1	0.74	0.63		
8	A1AG2 + CP + ITIH4 + LRG1 + ORM1	0.73	0.57		
9	CP + ITIH4 + LRG1 + ORM1 + TIMP1	0.74	0.56		
10	CP + ECM1 + IGHG2 + LRG1 + PON1 + SERPINA3 + CFH	0.78	0.67		
Consensus	CP+LRG1+ITIH4+TIMP1+ORM1			0.72	0.83
**Try 3 10-fold CV**	**Significant proteins selected into logistic regression models by stepwise selection**				
Fold	Predictive logistic regression models	Sub-Training (9/10 dataset 1)	Sub-Validation (1/10 dataset 1)	Training (dataset 1)	Validation (dataset 2)
1	CP + ITIH4 + LRG1 + ORM1 + TIMP1 + PRG4	0.78	0.56		
2	CP + IGHG2 + PON1 + SERPINA3	0.78	0.63		
3	CP + IGHG2 + PON1 + SERPINA3 + TIMP1	0.78	0.64		
4	CP + LRG1 + TIMP1 + PRG4	0.73	0.64		
5	CP + IGHG2 + PON1 + SERPINA3	0.75	0.81		
6	CP + LRG1 + PON1 + SERPINA3	0.75	0.80		
7	CP + FHR3 + ITIH4 + ORM1 + TIMP1	0.70	0.69		
8	CP + CTSD + FGG + IGHA2 + ITIH4 + SERPINA3 + TIMP1 + PLTP + FCGBP	0.79	0.43		
9	A1AG2 + CP + ITIH4 + LRG1 + ORM1	0.74	0.55		
10	CP + IGHG2 + LGALS3BP + PON1 + SERPINA3 + PRG4	0.77	0.74		
Consensus	CP+SERPINA3+TIMP1+PON1			0.74	0.83
**Try 1 8-fold CV**	**Significant proteins selected into logistic regression models by stepwise selection**				
Fold	Predictive logistic regression models	Sub-Training (9/10 dataset 1)	Sub-Validation (1/10 dataset 1)	Training (dataset 1)	Validation (dataset 2)
1	CP + IGHG2 + PON1 + SERPINA3 + VWF	0.7518	0.7929		
2	A1AG2 + CP + ITIH4 + LRG1 + ORM1 + TIMP1	0.7232	0.7337		
3	CP + MMRN1 + PON1 + SERPINA3	0.7569	0.6509		
4	CP + IGHG2 + PON1 + SERPINA3 + PRG4	0.7997	0.5799		
5	CP + FN1 + ITIH4 + LRG1 + SERPINA3 + TIMP1	0.7513	0.5417		
6	CP + ITIH4 + LRG1	0.738	0.5139		
7	CP + MMRN1 + PON1 + SERPINA3	0.7424	0.7222		
8	CP + PON1 + SERPINA3	0.7491	0.6458		
Consensus	CP+PON1+SERPINA3+LRG1+ITIH4			0.7528	0.8318
**Try 2**	**Significant proteins selected into logistic regression models by stepwise selection**				

**8-fold CV**					
Fold	Predictive logistic regression models	Sub-Training (9/10 dataset 1)	Sub-Validation (1/10 dataset 1)	Training (dataset 1)	Validation (dataset 2)
1	CP + ITIH4 + LRG1 + MMRN1	0.7377	0.5562		
2	CP + IGHG2 + LRG1 + PON1 + SERPINA3	0.7998	0.5444		
3	CP + ITIH4 + LRG1	0.7047	0.7633		
4	ECM1 + IGHG2 + LRG1 + MMRN1 + SERPINA1 + LUM	0.7725	0.6982		
5	CP + ITIH4 + LRG1 + TIMP1	0.7066	0.7431		
6	CP + MMRN1 + PON1 + SERPINA3	0.741	0.7778		
7	APOB + CP + CTSD + IGHG2 + PON1 + SERPINA3 + LUM	0.8133	0.5278		
8	CP + ITIH4 + LRG1	0.7327	0.5417		
Consensus	CP+PON1+SERPINA3+LRG1+ ITIH4+MMRN1+IGHG2			0.7691	0.8516

**Try 3 8-fold CV**	**Significant proteins selected into logistic regression models by stepwise selection**				
Fold	Predictive logistic regression models	Sub-Training (9/10 dataset 1)	Sub-Validation (1/10 dataset 1)	Training (dataset 1)	Validation (dataset 2)
1	CP + IGHG2 + LGALS3BP + PON1 + SERPINA3 + PRG4	0.7952	0.6095		
2	CP + MMRN1 + PON1 + SERPINA3	0.742	0.7041		
3	CTSD + ECM1 + ITIH4 + LRG1 + ORM1 + SERPINA1 + VTN	0.777	0.4379		
4	CP + FHR3 + PON1 + SERPINA3 + TIMP1 + SERPINA7 + KNG1	0.7811	0.5385		
5	CP + ITIH4 + LRG1 + MMRN1	0.7151	0.6458		
6	A1AG2 + CP + ITIH4 + LRG1 + ORM1	0.7322	0.5972		
7	ATRN + CP + IGHG2 + PON1 + SERPINA1 + SERPINA3 + TIMP1	0.8201	0.5972		
8	CP + ITIH4 + LRG1 + MMRN1 + PROC	0.7216	0.7361		
Consensus	CP+PON1+SERPINA3+LRG1+ ITIH4			0.7528	0.8318

Appendix Figure S1

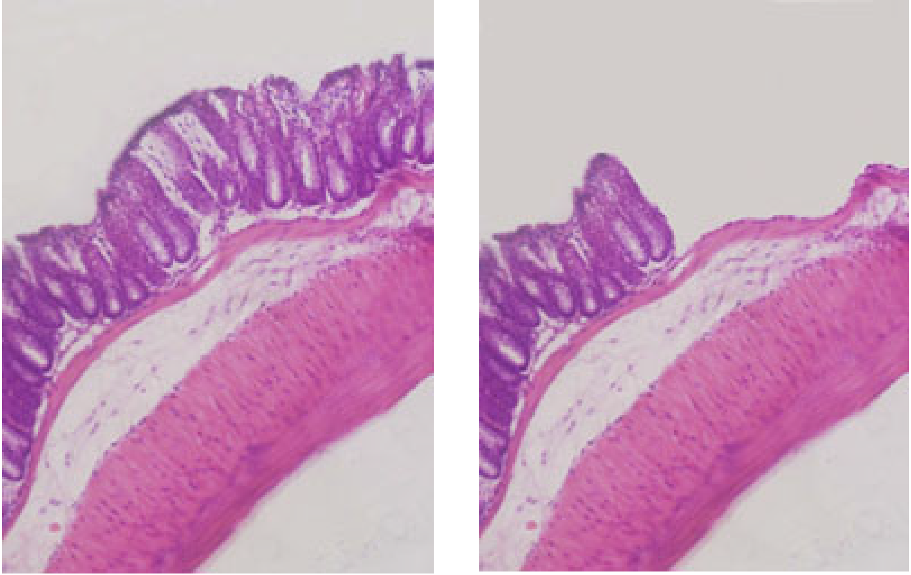

Appendix Figure S2

Logistic regression model

$$\ln \frac{\pi(\text{subject}_i)}{1-\pi(\text{subject}_i)} = -15.146 + 0.730 \times CP_i - 1.063 \times PON1_i + 0.686 \times SERPINA3_i + 0.374 \times LRG1_i + 0.413 \times TIMP1_i$$

$\pi(\text{subject}_i)$: the probability of CRC for ith subject

CP_i : the estimated log2 intensity of CP for ith subject
$PON1_i$: the estimated log2 intensity of PON1 for ith subject
$SERPINA3_i$: the estimated log2 intensity of SERPINA3 for ith subject
$LRG1_i$: the estimated log2 intensity of LRG1 for ith subject
$TIMP1_i$: the estimated log2 intensity of TIMP1 for ith subject

Standard errors of parameters based on logistic regression model fit

logistic model	estimate (parameter)	standard error of parameter
CP	0.7303	0.2307
PON1	-1.0628	0.286
SERPINA3	0.6864	0.2925
LRG1	0.3737	0.4134
TIMP1	0.4134	0.3838

Appendix Figure S3

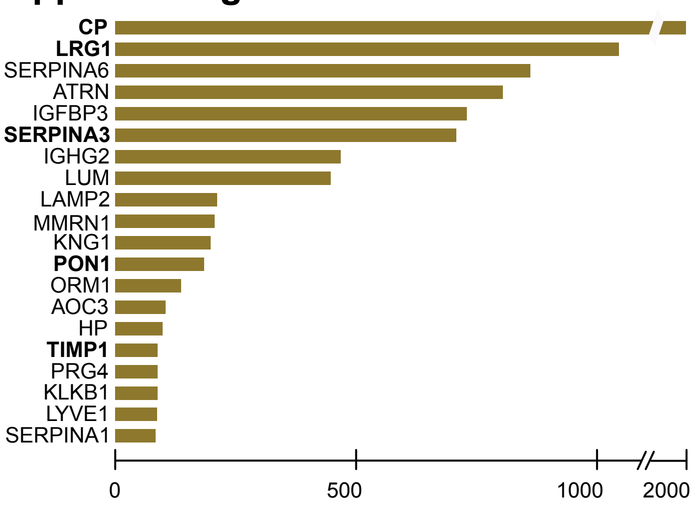

Appendix Figure S4

A

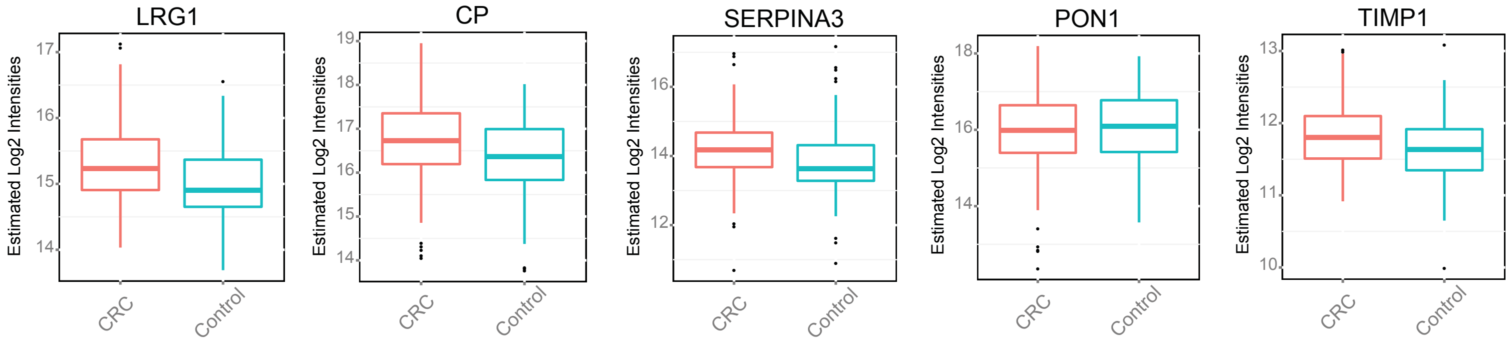

B

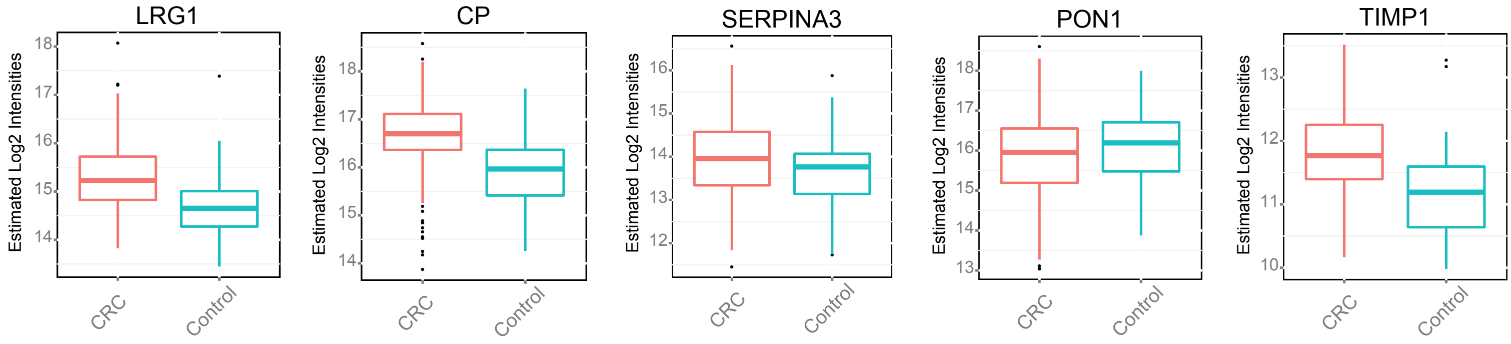

Appendix Figure S5

A

Sub-control group evaluation

	CRC vs all controls			**Pre-lesions** (Hyperplastic polyps & non-advanced adenomas)	**No lesions** (Negative tests)
fold 1	specificity	sensitivity	accuracy	specificity	specificity
cutoff=0.361	0.5	0.8	0.64	0.5	0.5
**fold 2**	specificity	sensitivity	accuracy	specificity	specificity
cutoff=0.401	0.6	0.85	0.73	0.57	0.62
fold 3	specificity	sensitivity	accuracy	specificity	specificity
cutoff=0.561	0.7	0.45	0.58	0.71	0.69
fold 4	specificity	sensitivity	accuracy	specificity	specificity
cutoff=0.334	0.32	0.75	0.54	0.17	0.38
fold 5	specificity	sensitivity	accuracy	specificity	specificity
cutoff=0.55	0.9	0.55	0.72	0.83	0.92

B

Additional control cohort of subjects with advanced adenomas

n	50
gender: female/male	21/29
median age: years (25 - 75 % quantiles)	65 (58.5 - 69)

Class prediction evaluation

Specificity	0.54

Appendix Figure S6

LRG1

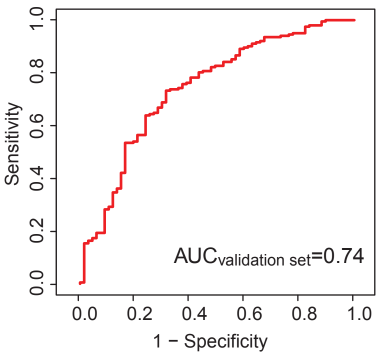

Validation statistics

Threshold	Specificity	Sensitivity	Accuracy
0.448	0.69	0.69	0.69

CP

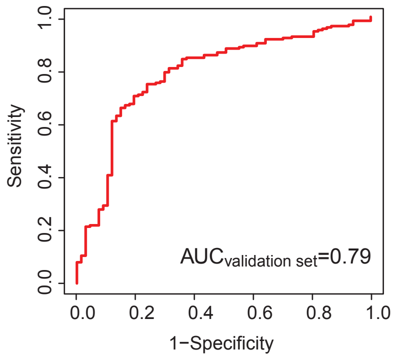

Validation statistics

Threshold	Specificity	Sensitivity	Accuracy
0.511	0.88	0.61	0.68

SERPINA3

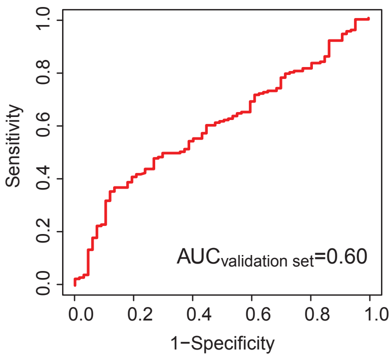

Validation statistics

Threshold	Specificity	Sensitivity	Accuracy
0.471	0.40	0.66	0.59

PON1

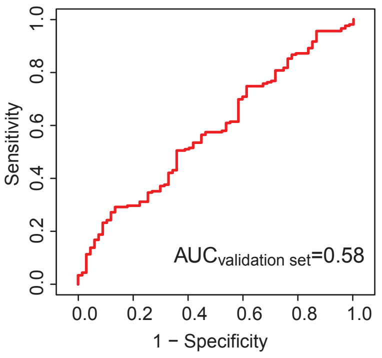

Validation statistics

Threshold	Specificity	Sensitivity	Accuracy
0.494	0.51	0.57	0.56

TIMP1

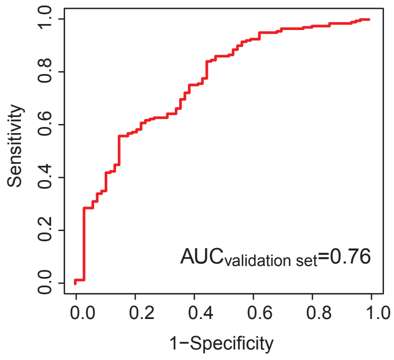

Validation statistics

Threshold	Specificity	Sensitivity	Accuracy
0.556	0.89	0.37	0.50

Appendix Figure S7

Prediction **without** age

fold	selected proteins	sub-training	sub-validation
1	CP + LRG1 + PON1 + SERPINA3	0.75	0.75
2	CD44 + CP + ITIH4 + LRG1 + ORM1	0.73	0.55
3	CP + HP + ITIH4 + ORM1 + TIMP1 + PROC	0.74	0.58
4	LRG1 + MMRN1 + ORM1 + PRG4	0.75	0.61
5	CP + PON1 + SERPINA3 + TIMP1	0.75	0.73
6	CP + CTSD + IGHG2 + PON1 + SERPINA3	0.76	0.72
7	CP + IGHG2 + PON1 + SERPINA3 + TIMP1	0.77	0.68
8	CP + ECM1 + PON1 + SERPINA3	0.77	0.43
9	CP + IGHA2 + IGHG2 + LGALS3BP + PON1 + SERPINA3 + PRG4	0.79	0.74
10	CP + FHR3 + FN1 + ITIH4 + LRG1 + TIMP1	0.75	0.54
≥5 folds	**CP+PON1+SERPINA3+LRG1+TIMP1**	0.75	
	**validation set**	0.84	

Prediction **with** age

fold	selected proteins	sub-training	sub-validation
1	CP + PON1 + SERPINA3 + THBS1 + Age	0.78	0.72
2	CP + ITIH4 + LRG1 + Age	0.75	0.88
3	CP + HP + ITIH4 + LRG1 + PROC + Age	0.78	0.57
4	MMRN1 + ORM1 + HP + PRG4 + Age	0.76	0.72
5	CP + ECM1 + FN1 + PON1 + SERPINA3 + TIMP1 + CD44 + Age	0.78	0.71
6	CP + IGHG2 + PON1 + SERPINA3 + Age	0.80	0.77
7	CP + IGHG2 + PON1 + SERPINA3 + Age	0.80	0.73
8	CP + ECM1 + PON1 + SERPINA3 + Age	0.79	0.58
9	CP + IGHG2 + LGALS3BP + PON1 + SERPINA3 + CD44 + PRG4 + Age	0.83	0.67
10	CP + FN1 + HP + ITIH4 + LRG1 + Age	0.77	0.69
≥5 folds	Age+CP+PON1+SERPINA3		
	**Age+CP+PON1+SERPINA3+LRG1+TIMP1**	0.78	
	**validation set**	0.89	

Parameters of logistic model with age

	estimate	std. error	z value	Pr(>|z|)
(Intercept)	-13.911	6.180	-2.251	0.024 *
Age	0.066	0.020	3.247	0.001 **
CP	0.623	0.240	2.627	0.009 **
PON1	-1.060	0.301	-3.517	0.000 ***
SERPINA3	0.738	0.307	2.407	0.016 *
LRG1	0.274	0.356	0.770	0.441
TIMP1	0.159	0.395	0.402	0.688

Signature's performance without/with age

Training set:

AUC difference=0.7806-0.7521=0.0285
p-value=0.1502

Validation set:

AUC difference=0.8391-0.8914=0.0523
p-value=0.0035

Appendix Figure S8

A

	AUC					
	**CEA**		**Signature**		**Signature + CEA**	
Fold	Sub-train	Sub-valid	Sub-train	Sub-valid	Sub-train	Sub-valid
1	0.506	0.643	0.840	0.886	0.856	0.900
2	0.499	0.682	0.858	0.743	0.870	0.743
3	0.542	0.624	0.856	0.752	0.865	0.790
4	0.537	0.414	0.868	0.677	0.876	0.707
5	0.519	0.504	0.843	0.865	0.856	0.879
6	0.522	0.534	0.846	0.842	0.859	0.827
7	0.524	0.481	0.838	0.902	0.851	0.895
8	0.524	0.491	0.839	0.947	0.853	0.939
9	0.523	0.526	0.842	0.886	0.856	0.912
10	0.505	0.667	0.843	0.886	0.858	0.886

B

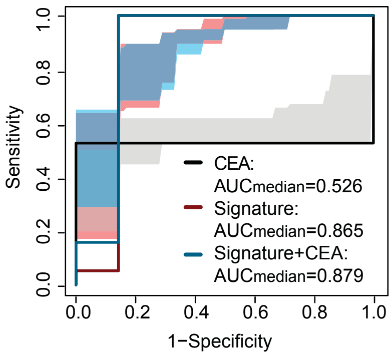

Signature *vs* CEA
Paired t-test:
mean difference=0.282
p-value=5.544e-05
95% CI=(0.192, 0.372)

Signature *vs* Signature + CEA
Paired t-test:
mean difference= -0.009
p-value=0.143
95% CI=(-0.022, 0.004)
