# Supplementary material for: Prediction of colorectal cancer diagnosis based on circulating plasma proteins
Source: EMBO Mol Med. 2015 Aug 7;7(9):1166–78. doi: 10.15252/emmm.201404873 (PMC4568950; doi:10.15252/emmm.201404873)
Supplement: Supplementary file 5 [file emmm0007-1166-sd5.pdf]

Prediction of colorectal cancer diagnosis based on circulating plasma proteins

Silvia Surinova, Meena Choi, Sha Tao, Peter J. Schüffler, Ching-Yun Chang, Timothy Clough, Kamil Vysloužil, Marta Khoylou, Josef Srovnal, Yansheng Liu, Mariette Matondo, Ruth Hüttenhain, Hendrik Weisser, Joachim M. Buhmann, Marián Hajdúch, Hermann Brenner, Olga Vitek, Ruedi Aebersold

Corresponding author: Ruedi Aebersold, ETH Zurich

Review timeline:

Submission date:	18 November 2014
Editorial Decision:	09 January 2015
Revision received:	11 May 2015
Editorial Decision:	18 June 2015
Revision received:	01 July 2015
Accepted:	07 July 2015

Transaction Report:

(Note: With the exception of the correction of typographical or spelling errors that could be a source of ambiguity, letters and reports are not edited. The original formatting of letters and referee reports may not be reflected in this compilation.)

Editor: Roberto Buccione

1st Editorial Decision 09 January 2015

Thank you for the submission of your two back to back manuscripts EMM-2014-04873, "Prediction of colorectal cancer diagnosis based on circulating plasma proteins" and EMM-2014-04874, "Non-invasive prognostic and predictive protein biomarker signatures associated with colorectal cancer" to EMBO Molecular Medicine. We have now heard back from the three Reviewers whom we asked to evaluate your manuscript.

We are sorry that it has taken longer than usual to get back to you on your manuscript. In this case we experienced some difficulties in securing three appropriate reviewers (due also to the heavier than usual burden), to whom I eventually granted a bit more time than usual. Obviously, the overlap with the holiday season did not help to speed things up.

You will see that the three Reviewers, albeit with varying degrees of enthusiasm, are globally supportive of your work but raise important concerns, which all considered, prevent us from considering publication at this time.

I will not dwell into much detail, and just mention a few main points. In my opinion, two main significant issues emerge, that are consistent across the reviewer panel and valid for both manuscripts. The first is that the clinical relevance of your findings is not convincingly demonstrated and that more experimentation is required to that effect. The second, connected, issue is that the potential advantage of your diagnostic and prognostic approaches with respect to the commonly accepted practices is not shown and must be convincingly explored, not merely

discussed. The Reviewers offer many approachable strategies to address these issues experimentally.

To mention one specific point, you will also see that two Reviewers are very critical of the rationale and usefulness of the KRAS signature in Ms. EMM-2014-04873.

The Reviewers also list other items for both manuscripts, which require your action.

In conclusion, while publication of the paper cannot be considered at this stage, given the potential interest of your findings and the fact that the Reviewers, although critical, were globally positive, we have decided to give you the opportunity to address the above concerns.

We are thus prepared to consider substantially revised submissions, with the understanding that the Reviewers' concerns must be fully addressed with additional experimental data where appropriate and that acceptance of the manuscripts will entail a second round of review. Please note that this should not be considered a commitment to publish on our side and that the two main issues raised as mentioned above, are very important ones for our title and should be satisfactorily addressed for us to consider publication. Also, the final decisions on the two manuscripts will be independent of each other.

Please note that it is EMBO Molecular Medicine policy to allow a single round of revision only and that, therefore, acceptance or rejection of the manuscripts will depend on the completeness of your responses included in the next, final versions of the manuscripts.

As you know, EMBO Molecular Medicine has a "scooping protection" policy, whereby similar findings that are published by others during review or revision are not a criterion for rejection. However, I do ask you to get in touch with us after three months if you have not completed your revision, to update us on the status. Please also contact us as soon as possible if similar work is published elsewhere.

I will be sending the same decision letter for both manuscripts so that you will be able to later provide separate rebuttals and revisions.

I look forward to seeing your revised manuscripts in due time.

***** Reviewer's comments *****

Referee #1 (Comments on Novelty/Model System):

This is the first comprehensive discovery and validation of a protein signature for colorectal cancer by high end mass spectrometry-based proteomics (shot-gun and targeted) in a substantial series of samples. All high quality analyses.

Regarding medical impact, it remains to be seen whether this plasma signature would outperform stool-based testing using the fecal immunochemical test.

Referee #1 (Remarks):

Surinova et al., describe the development of a protein biomarker panel for colorectal cancer detectable in plasma.The manuscript is impressive with respect to technical level of experiments as well as the sequence of experiments of glycoprotein discovery in patient tissue, via screening in blood to training/validation proof of concept signature development by targeted mass spectrometry in a substantial cohort of clinical samples. The study design and the number of samples tested is good, the manuscript is well-written and experiments are overall clearly described. The statistics used for signature identification is solid.

The 5 protein panel present in the diagnostic paper shows potential for future clinical application,

like for screening or disease monitoring in advanced disease. However the clinical application of this minimal invasive test should be further tested in a prospective screening cohort next to other non-invasive tests such as FIT or Cologuard test (followed up by colonoscopy) or for disease monitoring next to the currently used CEA test.

Comments/ questions:
In the introduction and discussion section, the traditional guaiac-based FOBT is presented as the current CRC detection procedure. This is incorrect since the current standard of care is either the immunologic FOBT test (FIT) or the recently FDA approved Cologuard test. Also recent studies show compliance rates of 73% for stool testing (Am J Gastroenterol. 2014 Aug;109(8):1257-64). Therefore the introduction should be modified and last paragraph in the discussion should be updated.

How does the 5-protein signature performs for diagnosing colorectal cancer in comparison to CEA?

The term 'benign subjects' is unclear; it should be subjects with benign lesions.

In the training cohort subjects with non-advanced adenomas are combined with subjects with hyperplastic polyps and no lesions. This is correct form a screening point of view, but did the authors check if the biomarker panel behaves the same for the subjects with or without adenomas? Also in the validation cohort the adenomas in the control group should be specified as advanced or non-advanced.

In the validation cohort the age distribution of the control group varies per condition, the largest subgroup being donors which are substantially younger that the patients. This may lead to a bias resulting from the age differences of the controls versus cases, which is an issue that should be addressed.

The SRM assay was initially developed for the 300+ protein candidates from the discovery, literature and other experiments and tested in the screening phase. The selection choices for inclusion in the targeted assay need to be more explicit. How many proteins were assayed in total (so including the non-consistent proteins)? Also what were the criteria for the proteins to fail this phase and for the 88 proteins to be reported as consistently detected and quantified? This information should be reported in the paper.

The link between glycoproteins (supplementary table S3) and glycopeptides in the targeted assay (Peptide atlas data screening and validation sets) is not clear. Supplementary table S3 should be augmented with the peptide sequences included in the targeted assay for each protein. Supplementary table S4 is missing from the uploaded data.

Referee #2 (Remarks):

The article by Surinova et al., "Prediction of colorectal cancer diagnosis based on circulating plasma proteins" describes an in-depth characterization of colorectal cancer (CRC) glycosylated proteins and their identification in blood of patients with CRC and healthy individuals with the goal of selecting CRC's protein markers. The authors first identified glycosylated proteins from CRC samples by glyco-selective enrichment and subsequent mass spectrometric analysis. Later, many of these proteins were detected and quantified in blood by SRM-based approach. Extensive statistical analysis led the authors to the conclusion that differential quantitation of 5 selected proteins in patient's blood is enough to accurately predict presence of CRC, thus offering a minimally invasive approach for CRC detection.

Major Issues:

1. The main criticism is the way the negative control group was analyzed and the errors associated with the measurements. The two main parameters of any clinical assay are sensitivity and

specificity. Comparison of CRC and healthy groups addresses mostly sensitivity, while for specificity a different set of experiments is required. Extensive comparison with different diseases has to be performed in order to conclude that the marker is indeed specific for the studied condition.

2. Since the authors mentioned in discussion that the fecal occult blood test (FOBT) is the most commonly employed non-invasive test for CRC, the authors should perform a comparison of the accuracy and sensitivity of FOBT and plasma biomarker signature.

3. As shown in the manuscript, the accuracy of current plasma biomarker signature for CRC is 72%. Could the authors also discuss how to improve their signature's accuracy?

4. The negative control group was heterogeneous and contained two subgroups: healthy and benign. Our guess is that by benign, the authors meant patients with non-cancer colon diseases which they described in the Materials and Methods. It appears that samples from all of those conditions were treated as one negative control group, thus eliminating the possibility of addressing specificity of the proposed biomarkers. For concluding that there are quantitative differences between CRC and, for example, Crohn's disease one has to compare samples from CRC and Crohn's disease, not CRC and mixture of Crohn's disease, benign tumors and healthy individuals.

5. The "benign" group was the smallest, numbering only 17 (healthy was 50 and CRC was 200) in the validation cohort. Since the "benign" group was heterogeneous and the number of samples from separate conditions is only a fraction of these 17 the question of specificity could not be addressed. To demonstrate this issue: two of the proposed biomarkers are well known acute phase proteins (ceruloplasmin and serpin A3) and were shown to be elevated in Crohn's disease. Additionally, we have found reports that TIMP1 (Am J Pathol. Apr 2003; 162(4): 1355-1360) and LRG1 (Inflamm Bowel Dis. 2012 Nov; 18(11):2169-79) are upregulated in Crohn's disease, while PON1 might be down regulated (Free Radic Biol Med. 2007 Sep 1;43(5):730-9). This is exactly the pattern described in this work, suggesting that the proposed five-plex assay may not be able to discern between CRC and Crohn's disease.-

6. All five proposed biomarkers have remarkably low fold changes (Figure 2, Step 4) - the highest being 1.6 for ceruloplasmin, the lowest 0.8 for PON1. In order to rely on such a small change the variation of concentrations of all of these five proteins between different people must be exceptionally low. There is no description of standard error associated with the measurements of individual candidate markers in the current article. However, Figure 3d depicts dependence levels of LRG1 and PON1 on the tumor sizes. It could be deduced from the image that the median protein levels between the smallest and the largest tumor sizes is about 1 on the log2 scale, i.e. it is about two-fold. This immediately suggests that the variation in levels of LRG1 and PON1 (2-fold) in the study sample alone are larger not only than the listed errors in Figure 2, Step 4 (0.03 and 0.06 correspondingly), but also than the proposed diagnostic fold changes themselves (1.4 and 0.8 correspondingly). It would be informative to have a box plot of healthy control group shown to get an idea of error distribution between the studied datasets.

7. It is not clear what is listed in the parenthesis in Figure 2, Step 4. If it is standard deviation then the authors should show how calculation of standard deviation of an average fold change has been performed since one can anticipate much larger variation by looking at Figure 3d.

Referee #3 (Remarks):

In this submission the Abersold laboratory take a mass spec based approach to discover a set of proteins in the blood that might act as new markers for the diagnosis of Colon cancer.

At present the FOBT test is used which is a relatively cheap and easy test though specificity of this is only approximately 50%. Following a positive FOBT then patients received endoscopy. One key question I feel not addressed in this manuscript is how clinically applicable this panel of markers will be (currently sensitivity it seems is 70% and selectivity of nearly 80%)?. Could this really be applied or is this another set of biomarkers that will not go beyond the first publication? That said this data here has been performed well and going from the tissue to markers from the blood is an

impressive amount of work. Also the size of the cohorts examined by this method is impressive.

Minor comments
1. LRG1 has previously been suggested as an early detection biomarker (it has been shown in APC PIRC rats for example, this should be cited). It is important for the authors to show how their data is better than a single marker such as LRG1.
2. CEA specificity and selectivity (when there is over 5ng/ml) appears to very similar to the new set of markers suggested here. Given this is a common and easy test, the authors should explain why their marker set is better (if is?). Again this is important as the key to the paper is whether this really is a viable set of markers that could be used in the clinic to diagnose CRC early.

1st Revision - authors' response 11 May 2015

Diagnostic paper

Referee #1 (Comments on Novelty/Model System):

This is the first comprehensive discovery and validation of a protein signature for colorectal cancer by high end mass spectrometry-based proteomics (shot-gun and targeted) in a substantial series of samples. All high quality analyses.

Regarding medical impact, it remains to be seen whether this plasma signature would outperform stool-based testing using the fecal immunochemical test.

Referee #1 (Remarks):

Surinova et al., describe the development of a protein biomarker panel for colorectal cancer detectable in plasma. The manuscript is impressive with respect to technical level of experiments as well as the sequence of experiments of glycoprotein discovery in patient tissue, via screening in blood to training/validation proof of concept signature development by targeted mass spectrometry in a substantial cohort of clinical samples.

The study design and the number of samples tested is good, the manuscript is well-written and experiments are overall clearly described. The statistics used for signature identification is solid.

The 5 protein panel present in the diagnostic paper shows potential for future clinical application, like for screening or disease monitoring in advanced disease. However the clinical application of this minimal invasive test should be further tested in a prospective screening cohort next to other non-invasive tests such as FIT or Cologuard test (followed up by colonoscopy) or for disease monitoring next to the currently used CEA test.

Comments/ questions:

In the introduction and discussion section, the traditional guaiac-based FOBT is presented as the current CRC detection procedure. This is incorrect since the current standard of care is either the immunologic FOBT test (FIT) or the recently FDA approved Cologuard test. Also recent studies show compliance rates of 73% for stool testing (Am J Gastroenterol. 2014 Aug;109(8):1257-64). Therefore the introduction should be modified and last paragraph in the discussion should be updated.

Both the introduction and discussion have been modified as suggested in the passages that concern the FOBT. In the discussion we now emphasize that patient compliance is improving.

How does the 5-protein signature performs for diagnosing colorectal cancer in comparison to CEA?

In the clinic, CEA is not recommended for the detection of CRC. The previous version of the manuscript partially confirmed this, in that the protein biomarker signature did not have a different predictive ability when stratified by CEA clinical cutoff of 5ng/mL (former **figure 3e,** current **figure 4a**). To address the reviewer's question more directly, we now evaluated the predictive ability of CEA alone (employing the clinical cutoff) in the validation cohort, and compared it to the performance of the protein biomarker signature developed in this study. 32% of subjects with CRC were detected by CEA alone in our validation cohort. The overall accuracy was 49%, which was much lower than the 72% accuracy of the protein biomarker signature presented here. We have made a new **figure 4** focusing on the CEA analysis on the validation cohort, where we added a ROC curve of CEA classification and overlaid it with the corresponding curve obtained by the protein signature (**figure 4b**). Finally, to further investigate whether CEA has any added impact on CRC detection beyond the protein signature, we generated a new model of CEA+protein signature within 10-fold cross validation. This analysis was performed on the validation cohort and the cross validation procedure was employed for the purpose of parameter estimation of the new combined model. **Figure S8** shows that there is no added benefit of this combination.

The term 'benign subjects' is unclear; it should be subjects with benign lesions.

We have corrected this term throughout the text.

In the training cohort subjects with non-advanced adenomas are combined with subjects with hyperplastic polyps and no lesions. This is correct form a screening point of view, but did the authors ***check if the biomarker panel behaves the same for the subjects with or without adenomas?*** *Also in the validation cohort the adenomas in the control group should be specified as advanced or non-advanced.*

We thank the reviewer for raising this very interesting point. We investigated it in much detail. Since the reviewer's question is about the control subjects from the training cohort, we employed 5-fold cross-validation and report the median performance to address this point. First, we examined the ability of the reported protein biomarker signature to predict pre-lesions (hyperplastic polyps and non-advanced adenomas, n=34) and no lesions (n=66). Both groups were predicted with similar accuracy, i.e. 57% of subjects with pre-lesions and 62% of subjects with no lesions were predicted correctly. This is also in line with the classification accuracy of the whole control group (n=100), which was 60%. It can therefore be concluded that the protein biomarker signature reported in this manuscript behaves very similarly for both groups.

To investigate this point further, we included a new set of advanced adenoma samples (n=50). These samples were collected and measured simultaneously with the training cohort, and appropriately randomized and normalized in order to avoid bias. Initially, we decided not to include these advanced adenoma samples as they represent intermediate lesions and our objective was to develop a signature for CRC detection. At this point, however, we thought they could be useful to address the reviewer's point further. We employed the new adenoma samples as an additional validation set, comprised from the subjects with advanced adenoma only and predicted the class of samples with our 5-protein biomarker signature developed on the training set. The accuracy of classification was 54%, which is similar with the overall as well as individual prediction of the former control groups. We could speculate that a slight drop in the predictive ability could point towards these subjects

representing an intermediate state of colorectal transformation. The new detailed analyses have been added into **figure S5**.

In the validation cohort, subjects with adenomas (n=4) have mid-to large dysplasia and therefore can be specified as having advanced adenomas. We have revised this in **Table 1** to specify that the adenoma samples in the validation set are advanced adenomas.

*In the validation cohort the **age distribution** of the control group varies per condition, the largest subgroup being donors which are substantially younger that the patients. This may lead to a bias resulting from the age differences of the controls versus cases, which is an issue that should be addressed.*

We agree with the reviewer that age is an important potential confounder for most biomedical investigations. Among the preliminary models considered in this work, we used models that adjusted the probability of the disease by age. The preliminary results showed that age is not a strong confounder in this case. To further study the raised concern, we performed the protein selection procedure within 10-fold cross-validation with or without age consideration for the respective individuals and found that very similar proteins were selected in both cases. The difference between the respective AUCs was 2.9% (bootstrap-based p-value=0.15). In the validation set, the difference between the model with or without age was 5.2% (bootstrap-based p-value=0.004). Although the obtained difference in AUCs is statistically significant, in both cases we obtained high AUC values (0.84 vs 0.89). We included these analyses in **figure S7**.

The SRM assay was initially developed for the 300+ protein candidates from the discovery, literature and other experiments and tested in the screening phase. The selection choices for inclusion in the targeted assay need to be more explicit. How many proteins were assayed in total (so including the non-consistent proteins)? Also what were the criteria for the proteins to fail this phase and for the 88 proteins to be reported as consistently detected and quantified? This information should be reported in the paper.

We clarified these details in the manuscript (**see page 3**). Specifically, the selection choices for inclusion as candidate were as follows: In the paired normal mucosa and tumor epithelia samples we characterized 303 proteins as differentially abundant, and supplemented these with 23 proteins that were only identified but not quantified in our discovery phase in the patient tissues. These proteins were reported in the literature as associated with cancer, and were therefore included in the screening phase in plasma. Additionally, we selected 5 proteins from other ongoing cancer biomarker studies in our laboratory for the same reasons as stated above. In total, 331 proteins were assayed in the screening phase. For these 331 proteins, we developed SRM assays and profiled them in plasma samples from 19 patients to determine their detectability. 88 of these proteins were consistently quantified in all of these samples. The reproducible detection and quantification of these proteins was the decisive criterion for their further evaluation in the third phase.

The link between glycoproteins (supplementary table S3) and glycopeptides in the targeted assay (Peptide atlas data screening and validation sets) is not clear. Supplementary table S3 should be augmented with the peptide sequences included in the targeted assay for each protein. Supplementary table S4 is missing from the uploaded data.

Table S4 has been previously in the supplementary information word document. We have now added the peptide sequences (available in PASSEL data repository) for easy access of the profiled peptide sequences and made the table an independent excel document file (**Table S4**). It shows which glycopeptides were monitored for the candidate glycoproteins.

© EMBO

Referee #2 (Remarks):

The article by Surinova et al., "Prediction of colorectal cancer diagnosis based on circulating plasma proteins" describes an in-depth characterization of colorectal cancer (CRC) glycosylated proteins and their identification in blood of patients with CRC and healthy individuals with the goal of selecting CRC's protein markers. The authors first identified glycosylated proteins from CRC samples by

glyco-selective enrichment and subsequent mass spectrometric analysis. Later, many of these proteins were detected and quantified in blood by SRM-based approach. Extensive statistical analysis led the authors to the conclusion that differential quantitation of 5 selected proteins in patient's blood is enough to accurately predict presence of CRC, thus offering a minimally invasive approach for CRC detection.

Major Issues:

1. The main criticism is the way the negative control group was analyzed and the errors associated with the measurements. The two main parameters of any clinical assay are sensitivity and specificity.

Comparison of CRC and healthy groups addresses mostly sensitivity, while for specificity a different set of experiments is required. Extensive comparison with different diseases has to be performed in order to conclude that the marker is indeed specific for the studied condition.

We agree with the reviewer that the assessment of disease specificity of the biomarker is important. Our study aimed at identifying biomarkers that differentiate between the healthy and the diseased states. The characterization of the specificity of the disease would require a different experimental design, which would include groups with multiple other disease conditions, as well as clinical data that clearly define each homogeneous patient subgroup. It would also require a different statistical analysis of such data, which would not only differentiate between CRC and controls, but would also show that this trend is absent in other disease conditions.

Since the number of other conditions and of possible combinations of conditions can be extremely large such investigation would essentially become an open-ended quest. For example, by examining the known biological roles of the biomarker signature proteins, we noted that two of the five proteins, SERPINA3 and CP, belong to the acute-phase proteins that are regulated in response to an acute inflammatory response. We could hypothesize that these two proteins can also be regulated under other inflammatory conditions, e.g. Crohn’s disease or other malignancies. Adding data that directly address the important point raised by the reviewer is therefore neither compatible with the design of the present study, nor of a scope that can be managed by our research group.

To respond to this comment, the revised manuscript now discusses this point on **page 9**. We emphasize the importance of biomarker specificity, and clarify that this manuscript does not claim the specificity of the protein biomarker signature as compared to other conditions. We further clarify that the control groups were selected to represent the general population of the subjects at risk, and that to our best knowledge the controls do not carry any comorbidities. We will pursue the characterization of these biomarkers in our future work. Meanwhile, we believe that the comprehensive and sensitive nature of the data acquired on the CRC and healthy subjects alone as part of the project already constitute a valuable advance, and deserve being reported and made publically available. We hope that the reviewer agrees with that.

2. Since the authors mentioned in discussion that the fecal occult blood test (FOBT) is the most commonly employed non-invasive test for CRC, the authors should perform a comparison of the accuracy and sensitivity of FOBT and plasma biomarker signature.

This suggestion is indeed very important. Unfortunately, we cannot perform this analysis on the subjects of the validation cohort because these subjects (except the healthy group) represent a population with symptomatic gastrointestinal tract (GIT) conditions. These subjects were selected based on their symptoms and were therefore directly evaluated by endoscopy/colonoscopy and, if positive, underwent surgery. To perform the requested comparison in a meaningful way, both tests would need to be performed on the same cohort, which is not possible for the reasons outlined above. It will be interesting to conduct an independent future study, which would employ a cohort designed specifically for this purpose, and derived from a screening setting. A follow up evaluation of the protein biomarker signature as compared to different FOBT tests is currently in preparation by the Aebersold laboratory and the clinical collaborators. Such study will require the approval for selection a new patient cohort, and a new effort of sample collection followed by the experimental and statistical analysis. Unfortunately, this effort is beyond the scope of what can be done with the current manuscript.

3. As shown in the manuscript, the accuracy of current plasma biomarker signature for CRC is 72%. Could the authors also discuss how to improve their signature's accuracy?

The accuracy of the signature can be improved on both clinical and technological levels.

On the clinical level, in contrast to new therapeutic entities, new biomarkers showing non-inferiority to existing biomarkers can still be useful in clinical practice, in particular because they may add to the accuracy of the existing biomarkers. Ultimately, additional extensive clinical research is required to prove the clinical utility of this approach. In particular, the combination of existing screening tests (i.e. FOBT) and new biomarkers (i.e. the proposed biomarker signature) may well add extra benefit in clinical practice. This will certainly be the focus of our future work, where the benefit of such a combination will be evaluated for this signature and different forms of the FOBT test. As mentioned above, this analysis will necessitate the design, approval and recruitment of an appropriate cohort, and measurements of the proposed signature proteins and of the FOBT tests on the same samples. Another option is to study the populations of subjects to discover homogeneous sub-populations, where the biomarker signature is particularly effective. These subsets could be identified from our cohort to some extent, but more appropriately, such an approach will require studies of a larger size and with more clinical characteristics. Finally, serial measurements monitoring the signature proteins over time, e.g. annually or biannually, as is often done for FOBT to improve its accuracy, would also likely enhance the signature’s accuracy.

On the technological level, to reach a high accuracy of CRC detection, further work needs to employ the results of the present study to develop a clinical grade multiplexed assay(Carr, Abbatiello et al. 2014). Such work will be able to optimize the following aspects of the measurements: (i) perform SRM measurements of the signature proteins alone with longer acquisition times than the ones employed in the case of a 90-plex method. This will lead to higher signal to noise ratio, lower limit of detection and more accurate quantification; (ii) employ absolute quantification with very precisely quantified internal standards; and (iii) develop ELISA assays for the signature proteins. Hence, more precise measurements will eliminate noise and contribute to more accurate discrimination.

These points were added into the discussion (**see page 9-10**).

4. The negative control group was heterogeneous and contained two subgroups: healthy and benign. Our guess is that by benign, the authors meant patients with non-cancer colon diseases which they

described in the Materials and Methods. It appears that samples from all of those conditions were treated as one negative control group, thus eliminating the possibility of addressing specificity of the proposed biomarkers. For concluding that there are quantitative differences between CRC and, for example, Crohn's disease one has to compare samples from CRC and Crohn's disease, not CRC and mixture of Crohn's disease, benign tumors and healthy individuals.

Since points 4 and 5 are related, we provide a single answer below.

5. The "benign" group was the smallest, numbering only 17 (healthy was 50 and CRC was 200) in the validation cohort. Since the "benign" group was heterogeneous and the number of samples from separate conditions is only a fraction of these 17 the question of specificity could not be addressed. To demonstrate this issue: two of the proposed biomarkers are well known acute phase proteins (ceruloplasmin and serpin A3) and were shown to be elevated in Crohn's disease. Additionally, we have found reports that TIMP1 (Am J Pathol. Apr 2003; 162(4): 1355-1360) and LRG1 (Inflamm Bowel Dis. 2012 Nov; 18(11):2169-79) are upregulated in Crohn's disease, while PON1 might be down regulated (Free Radic Biol Med. 2007 Sep 1;43(5):730-9). This is exactly the pattern described in this work, suggesting that the proposed five-plex assay may not be able to discern between CRC and Crohn's disease.

Since points 4 and 5 are related, we provide a single answer below.

A combined response to points 4 and 5:

The control group of the validation cohort was selected to include healthy subjects with no lesions and subjects with benign conditions as outlined in Table 1. The control arm of the cohort was designed to contain more subjects without any conditions, and slightly fewer subjects with non-malignant conditions (i.e. proportions that are more closely representative of a screening risk population). The subjects with non-malignant conditions were included because they represent a relevant control group of subjects without malignant disease but with potential precursors, thereby representing a realistic population. Due to the high heterogeneity of the controls, the study did not have enough power to separately investigate each small subgroup. Since our goal was not to differentiate between these sub-groups in an individual manner, we do not claim that we can separate between CRC and other conditions, e.g. Crohn's disease.

To investigate this point further, we included a new set of advanced adenoma samples (n=50) that represent more advanced lesions with possibly more inflammation. These samples were collected and measured as part of the training cohort, appropriately randomized and normalized to avoid bias. Initially, we decided to not include these advanced adenoma samples as they represent intermediate lesions and our objective was to develop a signature for CRC detection. At this point, however, we thought they could be useful to address the reviewer's point further. We employed the new adenoma samples as an additional validation set, comprised from the subjects with advanced adenomas only and predicted the class of samples with the protein biomarker signature. The accuracy of classification was 54%, which is similar with the 60% overall classification rate of control groups. We could speculate that a slight drop in the predictive ability could be accounted by the intermediate state of colorectal transformation of these subjects with advanced adenomas. The new detailed analyses have been added into **figure S5**.

6. All five proposed biomarkers have remarkably low fold changes (Figure 2, Step 4) - the highest being 1.6 for ceruloplasmin, the lowest 0.8 for PON1. In order to rely on such a small change the variation of concentrations of all of these five proteins between different people must be

exceptionally low. There is no description of standard error associated with the measurements of individual candidate markers in the current article.

However, Figure 3d depicts dependence levels of LRG1 and PON1 on the tumor sizes. It could be deduced from the image that the median protein levels between the smallest and the largest tumor sizes is about 1 on the log2 scale, i.e. it is about two-fold. This immediately suggests that the variation in levels of LRG1 and PON1 (2-fold) in the study sample alone are larger not only than the listed errors in Figure 2, Step 4 (0.03 and 0.06 correspondingly), but also than the proposed diagnostic fold changes themselves (1.4 and 0.8 correspondingly). It would be informative to have a box plot of healthy control group shown to get an idea of error distribution between the studied datasets.

Since points 6 and 7 are related, we provide a single answer below.

7. It is not clear what is listed in the parenthesis in Figure 2, Step 4.

If it is standard deviation then the authors should show how calculation of standard deviation of an average fold change has been performed since one can anticipate much larger variation by looking at Figure 3d.

Since points 6 and 7 are related, we provide a single answer below.

A combined response to points 6 and 7:

We thank the reviewer for pointing out that the description was insufficiently clear. In response to this comment, we revised **figure 2** and the legends of Figure 2 and 3, and added **figure S2** and **S4**. We summarize these changes below.

- In attempt to give a better insight in the data, Figure 2 provided data on fold changes of each individual protein selected in the signature, separately for the training data set (Step 2) and for the validation data set (Step 4). The numbers accompanying the fold changes in the parentheses are not the standard deviations but the standard errors. The fold changes and the standard errors were estimated using a linear mixed model implemented in the software package MSstats (Choi, Chang et al. 2014), and transformed to the original scale as $FC_{original}=2^{log2FC}$. The standard errors were transformed to the original scale using delta method (Agresti 2012) as $SE_{original}$=SE log2 scale x $ln(2)2^{log2FC}$. A detailed description of the calculation and tables with values on both the original and the log2 scale are to be found in **supplementary methods**. Given that the study has a relatively large sample size, it is not surprising that the standard errors are relatively small. The revised legend of Figure 2 now provides details of the calculation. Moreover, **Figure S4** provides boxplots of log-intensities of each protein in the CRC and in the control group, separately for the training and the validation sets. The log-intensities of each protein are summarized over all the protein transitions using MSstats.
- At the same time, we would like to emphasize that the fold changes and the standard errors are reported in Step 2 and Step 4 of Figure 2 for descriptive purposes only. The proposed protein signature was not derived on the basis of these individual fold changes, but on the basis of a joint multivariate modeling of all the selected proteins in a logistic regression. The logistic regression was also used to report the predictive ability of the signature. It is often the case in multivariate models that individual proteins with moderate fold changes have a stronger predictive ability jointly, as a group. This is in fact the main insight underlying the multivariate modeling. To clarify that, the legend of Figure 2 now emphasizes that Step 1 reports the parameters of the multivariate logistic regression used to evaluate the predictive ability of the signature. **Figure S2** now reports the standard errors of these parameters based on the logistic regression model fit.

Referee #3 (Remarks):

In this submission the Aebersold laboratory take a mass spec based approach to discover a set of proteins in the blood that might act as new markers for the diagnosis of Colon cancer.

At present the FOBT test is used which is a relatively cheap and easy test though specificity of this is only approximately 50%. Following a positive FOBT then patients received endoscopy. One key question I feel not addressed in this manuscript is ***how clinically applicable this panel of markers will be*** *(currently sensitivity it seems is 70% and selectivity of nearly 80%)? Could this really be applied or is this another set of biomarkers that will not go beyond the first publication? That said this data here has been performed well and going from the tissue to markers from the blood is an impressive amount of work. Also the size of the cohorts examined by this method is impressive.*

The issue of clinical utility of the discovered signature is of course of critical importance. Here we present a diagnostic protein signature that was translated from tissue into blood specimen across three clinical cohorts. The two large cohorts were taken from different geographical settings, hence they were truly independent. The multiplexed measurements of 88 candidate markers targeted in a reproducible manner across more than 500 subjects is unprecedented to the best of our knowledge, and offers a precedent of data sets for the evaluation of CRC on the protein level. Therefore this study cannot be compared to the numerous studies reporting lists of biomarker candidates identified in a single small set of samples and most typically not verified further.

At this stage we present a biomarker signature evaluated on two cohorts with clinical promise. In contrast to new medical treatments, new biomarkers showing non-inferiority to existing biomarkers might still be useful and added to clinical practice. Only extensive clinical research will ultimately prove clinical utility of existing and new biomarkers. Moreover, the scientific data alone may not be sufficient to determine the eventual application of the signature in the clinic, as other factors such as cost per analysis, intellectual property issues, market size and more will also play a role. These factors are not considered in this manuscript. Therefore, there is a lot of future work that needs to be done, scientific and otherwise, to bring it closer to the clinic.

One of the first follow up studies would compare the proposed signature with FOBT. To be able to accomplish this, a cohort needs to be designed, approved and recruited with subjects that were tested with FOBT tests and whose plasma samples are available for the measurement of the biomarker signature. In addition, the clinical benefit of a combination of the existing (e.g. FOBT) and proposed biomarkers can be explored. Furthermore, similarly to the serial testing of FOBT, it might be ultimately beneficial for the overall performance of the biomarker signature to profile it longitudinally, e.g. annually or biannually.

While the present study clearly leaves a lot of room for improvement and for subsequent follow-up, we believe that this work pushes the boundary of a typical biomarker discovery project beyond what is typically done, especially with respect the size of the clinical cohort and with respect to the independent validation. We believe that this study deserves to be reported and to be made publicly available, and we hope that the reviewer agrees.

Minor comments

1. *LRG1 has previously been suggested as an early detection biomarker (it has been shown in APC PIRC rats for example, this should be cited). It is important for the authors to show how their data is better than a single marker such as LRG1.*

In response to this comment, **Figure S6** has been added with performance metrics of the individual signature proteins. CP, TIMP1, and LRG1 showed the highest areas under the ROC curve of the five proteins but none of the individual proteins reached the performance of the biomarker signature.

We also discuss other reports on CRC detection of the individual proteins, when available, with the focus on studies performed in larger clinical cohorts (**see the discussion, page 8-9**).

LRG1 has been recently assayed by ELISA and reported to have an increased fold change and predictive ability of CRC detection in a cohort of 58 subjects with CRC and 58 control subjects, and also in a pre-diagnosis cohort of 32 female subjects with CRC and 32 female control subjects (Ladd, Busald et al. 2012). The size of the abundance change between the groups and prediction performance were highly in accordance with our results.

PON1 has been examined in the context of the serum oxidative imbalance association with an increased risk of CRC in a cohort of 40 subjects with CRC and 39 controls. Similarly to our results, lower serum PON1 activities were found in CRC patients(Bulbuller, Eren et al. 2013).

TIMP1 has been assayed by ELISA in blood samples and found to have elevated levels in 179 CRC patients as compared to 225 neoplasm-free participants, and its predictive ability to discriminate between these groups was slightly lower to the one found in our cohort (Tao, Haug et al. 2012).

2. *CEA specificity and selectivity (when there is over 5ng/ml) appears to very similar to the new set of markers suggested here. Given this is a common and easy test, the authors should explain why their marker set is better (if is?). Again this is important as the key to the paper is whether this really is a viable set of markers that could be used in the clinic to diagnose CRC early.*

First, we would like to clarify that the graph in the **former figure 3e** showed the performance of the protein biomarker signature on stratified groups by CEA cut-off. This was to show that we can equally detect subjects with very low concentrations of CEA with our signature. To address this comment, we have made a **separate figure 4** focusing a CEA and the protein biomarker signature. We have added the performance of CEA alone and in combination with the signature (**figure 4b**). The result shows that CEA is not a good diagnostic marker. We also evaluated any potential added benefit of CEA to the protein biomarker signature. For this analysis, we made a new model of CEA + protein signature within 10-fold cross validation and the result in **figure S8** shows that there is no added benefit of CEA in addition to the signature.

References

Agresti, A. (2012). Categorical Data Analysis, Wiley.

Bulbuller, N., E. Eren, H. Y. Ellidag, O. Z. Oner, C. Sezer, O. Aydin and N. Yilmaz (2013). "Diagnostic value of thiols, paraoxonase 1, arylesterase and oxidative balance in colorectal cancer in human." Neoplasma ***60****(4): 419-424.*

Carr, S. A., S. E. Abbatiello, B. L. Ackermann, C. Borchers, B. Domon, E. W. Deutsch, R. P. Grant, A. N. Hoofnagle, R. Huttenhain, J. M. Koomen, D. C. Liebler, T. Liu, B. MacLean, D. R. Mani, E. Mansfield, H. Neubert, A. G. Paulovich, L. Reiter, O. Vitek, R. Aebersold, L. Anderson, R. Bethem, J. Blonder, E. Boja, J. Botelho, M. Boyne, R. A. Bradshaw, A. L. Burlingame, D. Chan, H. Keshishian, E. Kuhn, C. Kinsinger, J. S. Lee, S. W. Lee, R. Moritz, J. Oses-Prieto, N. Rifai, J.

*Ritchie, H. Rodriguez, P. R. Srinivas, R. R. Townsend, J. Van Eyk, G. Whiteley, A. Wiita and S. Weintraub (2014). "Targeted peptide measurements in biology and medicine: best practices for mass spectrometry-based assay development using a fit-for-purpose approach." Mol Cell Proteomics **13**(3): 907-917.*

Choi, M., C. Y. Chang, T. Clough, D. Broudy, T. Killeen, B. MacLean and O. Vitek (2014). "MSstats: an R package for statistical analysis of quantitative mass spectrometry-based proteomic experiments." Bioinformatics.

*Ladd, J. J., T. Busald, M. M. Johnson, Q. Zhang, S. J. Pitteri, H. Wang, D. E. Brenner, P. D. Lampe, R. Kucherlapati, Z. Feng, R. L. Prentice and S. M. Hanash (2012). "Increased plasma levels of the APC-interacting protein MAPRE1, LRG1, and IGFBP2 preceding a diagnosis of colorectal cancer in women." Cancer Prev Res (Phila) **5**(4): 655-664.*

*Tao, S., U. Haug, K. Kuhn and H. Brenner (2012). "Comparison and combination of blood-based inflammatory markers with faecal occult blood tests for non-invasive colorectal cancer screening." Br J Cancer **106**(8): 1424-1430.*

2nd Editorial Decision 18 June 2015

Thank you for the submission of your revised manuscript to EMBO Molecular Medicine. Apologies for the unusual delay but we experienced some difficulties obtaining the evaluations in a timely manner and also because I wished to consult further with the Reviewers as explained below.

We have now received the enclosed reports from the Reviewers that were asked to re-assess it. As you will see, while two Reveiwers are now satisfied, Reviewer 2 has a remaining concern on specificity that s/he feels has not been adequately addressed.

After internal discussion and further consultation we agreed that the Reviewer does raise a good point over the specificity of the test, in the sense that if for example colitis/inflammation causes certain markers of the signature to go up, then this could compromise the valididty of the markers as bio markers. On the other hand, it may well be that the other markers are not expressed anyway, and thus this would not represent a problem. In conclusion, I would ask you to please provide a rebuttal and introduce a cautionary statement to this effect in the manuscript. Needless to say, should you have further data/analysis to support your point, this would be benefeical. It is most likely that and editorial decision will be made on your next, final version.

Please also include the "The paper explained" and "For more information" sections within the manuscript and not as a separate document.

I look forward to reading a new revised version of your manuscript as soon as possible and in any case possibly within two weeks.
I will proceed with acceptance of the accompanying manuscript EMM-2014-04874 "Non-invasive prognostic protein biomarker signatures associated with colorectal cancer" in parallel with acceptance of this manuscript, once deemed ready.

Please submit your revised manuscript within two weeks. I look forward to seeing a revised form of your manuscript as soon as possible.

I look forward to reading a new revised version of your manuscript as soon as possible.

***** Reviewer's comments *****

Referee #1 (Remarks):

My comments have been addressed and I am more than satisfied with the responses and extra data added to further clarify and strengthen the manuscript.

Referee #2 (Remarks):

In the rebuttal the authors explicitly indicate that the specificity of the proposed assay cannot be assessed with the current set of negative controls and that neither it was a goal of the current study:

The characterization of the specificity of the disease would require a different experimental design, which would include groups with multiple other disease conditions, as well as clinical data that clearly define each homogeneous patient subgroup. It would also require a different statistical analysis of such data, which would not only differentiate between CRC and controls, but would also show that this trend is absent in other disease conditions.

...this manuscript does not claim the specificity of the protein biomarker signature as compared to other conditions.

Since our goal was not to differentiate between these sub-groups in an individual manner, we do not claim that we can separate between CRC and other conditions, e.g. Crohn's disease.

Addressing specificity of a candidate biomarker does not require testing all pathological conditions:

Since the number of other conditions and of possible combinations of conditions can be extremely large such investigation would essentially become an open-ended quest.

However, some conditions that could reasonably be expected to produce the same response should be tested. It appears that the authors realized that but intentionally avoided addressing the specificity issue due to the fact that two of the 5 proteins in the pentaplex are well known acute phase proteins:

...we noted that two of the five proteins, SERPINA3 and CP, belong to the acute-phase proteins that are regulated in response to an acute inflammatory response. We could hypothesize that these two proteins can also be regulated under other inflammatory conditions, e.g. Crohn's disease or other malignancies. Adding data that directly address the important point raised by the reviewer is therefore neither compatible with the design of the present study, nor of a scope that can be managed by our research group.

The data presented in the current study, especially the appearance of SERPIN3 and CP in the pentaplex, strongly suggested unspecificity of the proposed assay in respect to inflammatory conditions, like Crohn's disease and it is imperative to address this issue. Also, why is inclusion of (t.ex.) Crohn's disease data in the analysis is not "...compatible with the design of the present study...". This would just be a separate control and it could be treated just like any other negative control.

Referee #3 (Remarks):

The authors have done a good job in answering my questions in their rebuttal letter and in the manuscript. The clarification of the CEA data is particularly helpful.
I still have concerns whether this signature will be clinically applicable but as the authors themselves note this is something to be determined in the future. That said the size and scope of this study is impressive and will act as important resource and step to finding new biomarkers.

2nd Revision - authors' response 01 July 2015

We thank you for the review and comments and for allowing us to comment further on the specificity of the diagnostic signature in a second revision cycle.

Please find our rebuttal on this last remaining item outstanding for the manuscript below. We have addressed the valid concern of the reviewer by adding further text to the discussion of the manuscript (please see the 2nd, 6th, and 7th paragraph of the discussion).

We hope that you will now find our manuscript satisfactory for publication in *EMBO Molecular Medicine*.

Reply: The objective of the present study was to develop a biomarker assay for the non-invasive diagnosis of CRC. CRC is a heterogeneous disease where multiple cancer modalities contribute to its systemic demonstration that can be monitored in the blood circulation. Among the known cancer hallmarks, tumor-promoting inflammation is an enabling characteristic that incites and promotes carcinogenesis (Hanahan, 2011). It plays an important role at all stages of cancer progression. Given the complex characteristics of CRC, it is unlikely that a single protein marker would be powerful enough to capture this disease across a large set of patients. We have therefore included the most discriminating protein predictors into a multivariate signature that capture the different aspects of CRC as compared to healthy and benign controls in the circulation. The proteins comprising the diagnostic signature have been previously linked to CRC and play different functional roles (as detailed on page 8 and 9 of the discussion). We clearly show that these proteins, in combination, represent the most powerful pattern for CRC detection in a large number of subjects that likely have heterogeneous presentations of the disease.

The cancer-associated proteins within our signature contain both tumor-derived and systemic response proteins. We could speculate that since the proteins associated with a systemic inflammatory response have been reported to play an important role across all stages of cancer progression, it may well be that they demonstrate a rather stable upregulation across patients. This could explain why SERPINA3 and CP were prioritized into the signature and it may be indispensable to capture tumor-derived inflammation with these proteins in the multivariate signature.

SERPINA3 and CP can be upregulated in cancerous or non-cancerous inflammatory conditions. Crohn's disease (CD) and ulcerative colitis (UC) are the most common forms of inflammatory bowel disease (IBD). IBD uses C-reactive protein (CRP) as the main diagnostic marker. Anti-Saccharomyces cerevisiae antibodies (ASCA) and anti-neutrophil cytoplasmic antibody (ANCA) belong to the other two best-studied serum markers in IBD (Bennike, 2014). Other inflammatory proteins have not been validated for IBD detection.

The differences in inflammation between cancerous and non-cancerous inflammatory conditions are not well understood, also due to a lack of studies with enough power to assess differences in related conditions. Only a direct assessment of these proteins across large enough groups of CRC and IBD patients will expand our current understanding of inflammation in CRC as compared to IBD, and to that end any differences between Crohn's disease and UC. One study worth mentioning examined differences between UC progressors with or without dysplasia (i.e. an inflammatory condition that could transform into a cancerous state) and found their top candidates to be mitochondrial proteins, cytoskeletal proteins, proteins from the RAS superfamily, proteins relating to apoptosis, and metabolism (May, 2011).

The specificity of the signature for CRC is significantly mediated by TIMP1, PON1, and LRG1 - the three proteins in the signature previously associated with CRC more directly at the tumor site. Additionally, our own observations revealed that the abundance of PON1 and LRG1 in the circulation of CRC patients correlated with the tumor size in these patients, which points towards tumor-specific markers. TIMP1 was previously reported to yield diagnostic value for CRC detection in large cohorts (Holten-Andersen et al, 2002). Interestingly, in this study the authors measured

TIMP1 levels in IBD and found no significant differences in plasma TIMP1 levels between healthy donors and IBD.

In conclusion, a combination of proteins characterizing the different features of CRC, including inflammation, may provide a more powerful diagnostic test than when focusing on probable tumor-derived proteins alone. Although our study was not designed to address the signature specificity with respect to other inflammatory conditions at this stage, future evaluation of the diagnostic signature should assess the specificity of the signature in groups of IBD conditions. These sample groups will need to be large enough to perform this analysis at the required statistical power. The current study represents a major advancement in biomarker evaluation area by proteomics and sets a precedent in this respect. The main reason why these additional groups were not included at this stage, is the feasibility of such an investigation in an academic setting. At this point we have reported a multivariate signature capturing the most significant CRC demonstrations in the circulation with a signature comprised of a few proteins that can be further evaluated by mass spectrometry or immunoassays in additional cohorts ranging across CRC development and conditions predisposing or sharing certain aspects of demonstration.

References

Hanahan, Weinberg RA. Cell. 2011 Mar 4;144(5):646-74

Bennike et al. Biomarkers in inflammatory bowel diseases: current status and proteomics identification strategies. World J Gastroenterol. 2014 Mar 28;20(12):3231-44.

May et al. Investigating Neoplastic Progression of Ulcerative Colitis with Label-Free Comparative Proteomics. J Proteome Res. 2011 Jan 7; 10(1): 200–209.

Holten-Andersen et al. Total levels of tissue inhibitor of metalloproteinases 1 in plasma yield high diagnostic sensitivity and specificity in patients with colon cancer. Clin Cancer Res. 2002 Jan;8(1):156-64.

© EMBO
